# Study protocol for the Australasian Cerebral Palsy Musculoskeletal Health Network (AusCP MSK) prospective cohort study: early detection of musculoskeletal complications in young children with moderate to severe cerebral palsy (GMFCS III–V)

**DOI:** 10.1136/bmjopen-2024-095526

**Published:** 2025-04-30

**Authors:** Craig F Munns, Laura A Bentley, Roslyn N Boyd, Denise Brookes, Maddison J Taylor, Peter Pivonka, Natasha Nassar, Stewart G Trost, J Paige Little, Kylie Tucker, Joshua Burns, Leanne Sakzewski, Nadia Badawi, Robert S Ware, Tracy Comans, Kate L Willoughby, Simon Paget, AusCP MSK Collaborator Group, Stina Oftedal

**Affiliations:** 1 Queensland Cerebral Palsy and Rehabilitation Research Centre (QCPRRC), The University of Queensland, Brisbane, Queensland, Australia; 2 Children’s Health Queensland Hospital and Health Service, Brisbane, Queensland, Australia; 3 Queensland Paediatric Rehabilitation Service, Queensland Children’s Hospital, Brisbane, Queensland, Australia; 4 Medical Imaging and Nuclear Medicine, Queensland Children’s Hospital, Brisbane, Queensland, Australia; 5 Children’s Hospital at Westmead, Sydney, New South Wales, Australia; 6 The Australian E-Health Research Centre, CSIRO, Brisbane, Queensland, Australia; 7 Monash University, Melbourne, Victoria, Australia; 8 Hudson Institute and Monash University, Melbourne, Victoria, Australia; 9 Murdoch Children’s Research Institute, Melbourne, Victoria, Australia; 10 Sydney Children’s Hospitals Network, Sydney, New South Wales, Australia; 11 The Garvan Institute of Medical Research, Sydney, New South Wales, Australia; 12 The University of Sydney, Sydney, New South Wales, Australia; 13 University of Western Australia, Perth, Western Australia, Australia; 14 The University of Auckland, Auckland, New Zealand; 15 Starship Children’s Health, Auckland, New Zealand; 16 Liggins Institute, The University of Auckland, Auckland, New Zealand; 17 The University of Queensland, Brisbane, Queensland, Australia; 18 The Royal Children’s Hospital, Parkville, Victoria, Australia; 1 Child Health Research Centre, The University of Queensland, Brisbane, Queensland, Australia; 2 Queensland Cerebral Palsy and Rehabilitation Research Centre, The University of Queensland, Brisbane, Queensland, Australia; 3 Faculty of Engineering, Queensland University of Technology, Brisbane, Queensland, Australia; 4 Faculty of Medicine and Health, The University of Sydney, Sydney, New South Wales, Australia; 5 Faculty of Human Movement and Nutrition Sciences, The University of Queensland, Brisbane, Queensland, Australia; 6 Biomechanics & Spine Research Group, Queensland University of Technology, Brisbane, Queensland, Australia; 7 Faculty of Health, Medicine and Behavioural Sciences, The University of Queensland, Brisbane, Queensland, Australia; 8 Department of Epidemiology and Cancer Control, St Jude Children’s Research Hospital, Memphis, Tennessee, USA; 9 The University of Sydney, Sydney, New South Wales, Australia; 10 Griffith Biostatistics Unit, Griffith University, Brisbane, Queensland, Australia; 11 The Royal Children’s Hospital Melbourne, Parkville, Victoria, Australia; 12 Kids Rehab, Children’s Hospital at Westmead, Westmead, New South Wales, Australia

**Keywords:** Scoliosis, Clinical Protocols, Disabled Persons, Hip, Fractures, Bone

## Abstract

**Background:**

Cerebral palsy (CP) is the most common physical disability of childhood, affecting movement and posture, resulting from a neurological insult during pregnancy or the neonatal period. While the brain lesion is static, the musculoskeletal sequelae in CP are often progressive and lifelong, associated with pain and can impact the lives of children with CP, their families and the healthcare system. The Australasian Cerebral Palsy Musculoskeletal Health Network (AusCP MSK) study will conduct comprehensive, population-based surveillance of children with moderate to severe functional mobility limitations (Gross Motor Function Classification System (GMFCS) levels III–V) to explore the early biomarkers of, and interactions between, musculoskeletal complications related to CP, including hip displacement, scoliosis and skeletal fragility.

**Methods:**

The AusCP MSK study involves three cohorts of children. Cohort A (n=500) is a multicentre retrospective (3 years) and prospective (4 years) cohort study in children aged 4–9 years that will be implemented at five sites across Australia and New Zealand. Retrospective data will include clinical history, information on CP diagnosis and other investigations (previous X-rays and biochemistry). Primary prospective outcomes will involve measures of hip displacement (migration percentage, acetabular index, femoral head orientation, Hilgenreiner’s epiphyseal angle), scoliosis (Anteroposterior/Posteroanterior and lateral spine X-ray), skeletal fragility (Dual Energy X-ray Absorptiometry, peripheral quantitative computed tomography), motor function (GMFCS, Manual Ability Classification System (MACS) and Communication Function Classification System (CFCS)) and range of movement (lower limb and spine). Cohort B (n=4000) is a retrospective analysis of data to evaluate fractures in children up to 18 years of age with CP (GMFCS I–V) from the New South Wales (NSW)/Australian Capital Territory CP Registers linked with corresponding records from NSW administrative health data (n=3000), and a New Zealand cohort of linked data from the New Zealand Cerebral Palsy Register to the Accident Compensation Corporation data for fracture claims (n=1000). Cohort C (n=30) will cross-sectionally examine bone quality through a transiliac bone biopsy in children undergoing scheduled hip surgery. Relationships between early biomarkers, early brain structure and musculoskeletal complications will be explored using multilevel mixed-effect models.

**Ethics and dissemination:**

Ethical approval for this study was granted by Children’s Health Queensland Hospital and Health Service Human Research Ethics Committee, The University of Queensland Human Research Ethics Committee and the New Zealand Health and Disability Ethics Committee.

Research outcomes will be disseminated via scientific conferences and publications in peer-reviewed journals; to the National Bodies and Clinicians; and to people with CP and their families.

**Trial registration number:**

Australian New Zealand Clinical Trials Registry number: ACTRN12622000788774p

**STRENGTHS AND LIMITATIONS OF THIS STUDY**

- The study employs a prospective cohort design, allowing for the longitudinal assessment of musculoskeletal complications in children with moderate to severe cerebral palsy (CP).
- The study leverages multisource data linkage and imaging-based assessments across Australia and New Zealand enhancing population representativeness and generalisability.
- Possible risk of participant dropout and incomplete data across the study.
- Findings will be limited to children with moderate to severe CP (Gross Motor Function Classification System III–V) and results may not be generalisable to those with milder motor impairments.

## Introduction

 Cerebral palsy (CP) is the most common physical disability of childhood impacting 1 in 700 children in Australia.^1^ The condition affects the development of movement and posture, causing activity limitations, commonly attributed to non-progressive disturbances that occurred in the developing fetal or neonatal brain.^2^ The musculoskeletal sequelae in CP are progressive and life-long, extensively impacting the lives of children with CP, their families and the healthcare system and are often associated with significant pain.^3^

### Background to Musculoskeletal Abnormalities in Cerebral Palsy

The mechanical properties of bone size, shape and strength,^4 5^ are determined by genetics, bone growth and the forces applied to the bone by muscles and weightbearing.^6^ In children with CP, the initial static neurological insult leads to spasticity/dystonia and/or weakness, resulting in abnormal muscle forces on the developing skeleton, which impacts bone morphology and contributes to progressive hip displacement, scoliosis and skeletal fragility.^7^ These musculoskeletal alterations continue across the lifespan,^8^ contributing to reduced motor function, joint displacement (eg, hip displacement), increased joint pain, reduced habitual physical activity (HPA), increased sedentary behaviour, increased difficulty in care giving^9^ and decreased quality of life (QoL).^10^ The altered musculoskeletal development begins early in children with CP,^11^ resulting in muscles with reduced volume,^8 12^ with muscle fascicles that are stiffer,^13^ have reduced force production capacity,^11^ are thinner^14^ and shorter with longer tendons.^14 15^ The altered muscle activation patterns and reductions in muscle force, applied in abnormal directions, result in ongoing perturbations to skeletal development. In the hip, abnormal femoral head and acetabular development and coxa valga, all contribute to hip displacement.^16^ Increased hip adductor spasticity has also been considered as a contributing factor of hip displacement.^17^,^19^ At the spine, there is abnormal vertebral growth and reduced vertebral strength, which may contribute to scoliosis development.^20^ Reduced muscle force can also impact bone mass accrual throughout the skeleton, increasing the risk of fragility fracture.^21^ The precise age of onset of musculoskeletal complications, detailed knowledge of genetic predisposition, potential early biomarkers and complex interactions between the musculoskeletal complications of CP remain unclear.

### Hip displacement

Children with CP with moderate to severe mobility limitations (GMFCS levels III–V)^22^ have a high risk of progressive hip displacement with an incidence of approximately 35%.^23 24^ The pathophysiology of hip displacement in CP has been historically based on the spastic muscle model, which postulates that initial brain damage leads to spasticity characterised by muscle stiffness in the hip adductors and flexors, which pulls the femoral head out of the hip joint.^16^ While the spastic muscle model seems logical, there is evidence that hip displacement is unrelated to motor type, that is, there is no greater risk of hip displacement in children with spasticity than hypotonia.^23 24^ A more holistic model of hip displacement focuses on the negative features of CP as an upper motor neuron syndrome, with muscle weakness and limited weight bearing and walking function as causes of hip displacement.^16^ Hip abductor weakness and limited weight bearing/walking lead to abnormal joint forces, causing changes in the proximal femoral geometry (increased femoral neck anteversion and neck shaft angle) subsequently leading to hip displacement.^16 25^ When not treated early or effectively, displacement can lead to painful dislocation of the hip, with decreased function, increased carer burden and decreased QoL.^26 27^ A Victorian population-based study (n=323) recognised GMFCS as the strongest predictor of progression of hip displacement, defined as migration percentage (MP) on anteroposterior pelvic X-ray of greater than 30%, with increasing risk in children who are marginal ambulators GMFCS level III (41.3%); those non-ambulant but able to sit GMFCS IV (69%) and those unable to sit GMFCS V (90%).^24^ Based on the findings of these studies, a number of clinical guidelines for the systematic routine surveillance of hip displacement have been developed, including in Australia.^28^,^30^

Several longitudinal studies have demonstrated the effectiveness of population-based hip surveillance programmes on reduced rates of complete hip dislocation through early identification and timely referral for orthopaedic assessment and surgical management^31^,^33^; these do not, however, elucidate whether specific factors are protective against the development of progressive hip displacement for some children, such as increased habitual activity levels in children at GMFCS III, or floor mobility and standing function in those functioning at GMFCS IV.

There is no uniformly recognised treatment for prevention of hip displacement in children with CP, with interventions such as intramuscular Botulinum toxin A injections, Intrathecal Baclofen (ITB) and Selective Dorsal Rhizotomy (SDR) having limited effectiveness.^34^,^38^ Hip surgery (femoral osteotomy) is a well-recognised treatment for children with significant displacement or dislocation of the hips.^39^ Children with a hip MP of ≥30% are at higher risk of progressive hip displacement,^40^ and a hip with an MP ≥50% is at higher risk of dislocation.^41^ Surgical reconstruction has been demonstrated to be effective in improving pain and mobility in children with CP with marked hip displacement.^42^,^46^

### Scoliosis

Progressive spinal deformities, including scoliosis and kyphosis, are a major problem in children functioning at GMFCS levels IV and V, and can result in limitations to function and activity, cause pain and can be life-limiting due to respiratory compromise.^47^ Scoliosis is defined as a spinal curve of angle >10° as measured using the Cobb method.^48 49^ Similar to hip dysplasia, the incidence of scoliosis in children with CP has a direct correlation with GMFCS level.^50 51^ A Swedish prospective longitudinal study (n=1025), conducted within the follow-up healthcare programme and registry for children with CP (CPUP), reported the risk of severe scoliosis (Cobb angle >40°) at 10 years old was 2% for GMFCS III, 5% in GMFCS IV and 20% in GMFCS V. At 20 years, the prevalence of severe scoliosis (Cobb angle >40°) was 8% for GMFCS III, 35% for GMFCS IV and 75% for GMFCS V.^50^

Similarly, in an Australian cohort (n=292), a scoliosis (Cobb angle >10°) was found in 41% of individuals with CP and was directly related to increasing GMFCS level. Severe scoliosis (Cobb angle >40°) was most common in children functioning at GMFCS IV and V (18% and 48%, respectively). Curves were also more common in young people with more severe limitations in fine motor function and the presence of dystonia or mixed movement disorders.^52^ There is limited research that has explored protective factors for preventing scoliosis in children with CP. In addition, the relationship between hip displacement and scoliosis and the inter-related musculoskeletal complications are not well established in child populations.^49^ In adult populations with CP, the association between asymmetrical limited hip flexion (<90°), pelvic obliquity and clinical assessment of scoliosis is well established.^53^ In children with CP, postural asymmetry may play a role in increasing the likelihood of developing scoliosis, windswept and contracted hips.^54^ While the convexity of scoliosis has been associated with the high side of pelvic obliquity in young people with CP,^55^ not all who have a scoliosis and pelvic obliquity will develop significant hip displacement, and the natural history and aetiological mechanisms which underpin severe scoliosis in young people with CP are not clear. These studies highlight associations between hip abnormalities and scoliosis in children and adults with CP, however a causative relationship remains unclear. There are few prospective studies in children with CP that have investigated the early biomarkers of progression and the inter-relationship between hip displacement and scoliosis, confirmed by radiology, within the same cohort. Also, while guidelines for hip surveillance have been available in Australia since 2008,^29^ there is currently no national clinical guideline for the routine identification and surveillance of scoliosis.

To manage scoliosis, surgical intervention is considered the main treatment pathway,^56^ with non-surgical management including bracing and postural support in seating for prevention and slowing progression of minor curves. Bracing can improve sitting posture and trunk support, enabling better head, neck and upper limb control;^57 58^ however, bracing treatment can be difficult to prescribe and is poorly tolerated by young people with CP with significant dystonia or hypertonia, cognitive impairment or associated neurodiverse conditions due to the invasive and restrictive nature of casting and braces. Evidence of the effectiveness of bracing is mixed, with some demonstrating that bracing can slow curve progression, specifically in younger children with Cobb angles of less than 40°. ^57^,^59^ Other research, however, has suggested that for most children with CP braces are insufficient to reduce the requirement of surgical intervention,^58^ although in non-ambulatory individuals with scoliosis, optimising seating position has been identified as effective in increasing support and improving functional outcomes.^60^

### Skeletal fragility

Skeletal fragility is a significant complication of CP that worsens with disability and age.^61^ Skeletal fractures cause pain and result in diminished QoL and substantially increase costs of medical care as they can occur frequently and throughout the life span.^62^,^64^ Abnormal bone development during growth in children with CP increases the risk of pathological fracture that persists into adult life.^65^ The incidence of pathological fracture of the femur, tibia and humerus in children with CP is approximately 40%, with an annual fracture rate of 7%–10%.^66^ This has not, however, been evaluated in an Australian cohort. Although the overall incidence of fracture in children with CP is no greater than typically developing children (TDC), it is the mechanism of fracture (minimal or no trauma in children with CP), the fracture site (femur, tibia and humerus in CP as opposed to distal radius and ulna in TDC) and the associated morbidity that makes skeletal fragility a significant issue.^67^ The aetiology of reduced bone mass for age and reduced bone strength in children with CP is multifactorial. The primary factor is reduced mobility and associated reduced muscle force on bone.^66 68^ Other factors that can impact bone mass accrual include vitamin D deficiency, dietary calcium deficiency, undernutrition and low body weight, pubertal delay, and medications such as anticonvulsants, steroids and diuretics.^69 70^ Although Australian investigators have published consensus guidelines^71^ and undertaken studies on the prevention and treatment of skeletal fragility in children with CP,^72 73^ there is currently no predictive model to determine prognosis, best therapeutic actions or strategies to reduce the burden of fracture in children with CP.^74 75^ Improving our understanding of bone health would enable timely intervention to optimise bone strength, prevent complications of pain, pathological fracture and failure of orthopaedic devices. An important component of the AusCP Musculoskeletal Health Network (AusCP MSK) study is fully characterising the bone of children with CP through a combination of dual-energy X-ray absorptiometry (DXA), an *in vivo* peripheral quantitative CT (pQCT) and *ex vivo* bone mineral density distribution (BMDD).

Interventions designed to reduce skeletal fragility in children with CP include weight-bearing exercise, specifically including high ground reaction force or pull/strain on the skeleton over a prolonged period of time, such as vibration platforms.^76^ Positive treatment effects have been documented for weekly callisthenics programme^77^ and whole body vibration therapy.^78^ Current guidelines in CP recommend the treatment of fragility fractures with bisphosphonates.^71 73^ There is some contention, however, about the paucity of rigorous studies that examine the efficacy and safety of this approach.^72^

### Significance of the current study

The overarching goal of the AusCP MSK Network is to conduct comprehensive, population-based surveillance of children with CP to better understand the aetiology, age at onset, rate of progression and the clinical significance of musculoskeletal complications in CP as well as to identify therapeutic targets to inform prevention programmes for musculoskeletal complications. Musculoskeletal complications related to CP for non-ambulant children, including hip displacement, scoliosis and skeletal fragility are often severe and progressive, with a life-long impact. Currently in Australia, there are a number of guidelines in place to inform clinical practice in people with CP. These include the Australasian Cerebral Palsy Clinical Trials Network (Aus-CP-CTN) on early detection,^79^ early intervention^80^ and efficacy of interventions to improve functional outcomes.^81^ The Australian Hip Surveillance Guidelines for Children with Cerebral Palsy^28^,^30^ have been implemented to varying degrees in most states, however, there are no existing clinical guidelines or registries for spinal surveillance and skeletal fragility which this study aims to address. This has prevented the investigation of early biomarkers for, and the inter-relationship between, hip displacement, scoliosis and skeletal fragility. These knowledge gaps have hampered the development of timely and effective interventions aimed at either preventing or mitigating these musculoskeletal complications. Ultimately, early detection of musculoskeletal complications in children with CP, coupled with evidence-based preventative interventions, will reduce the impact of spine, hip and skeletal fragility disorders in children, adolescents and adults with CP. This will support people with CP to have the best possible musculoskeletal health, physical function and QoL, leading to a reduction in healthcare-related and societal costs. The AusCP MSK—MRF2015970 is prospectively registered at ACTRN: ACTRN12622000788774p and ACTRN12622000782730p.

## Methods and analysis

### Aims and hypotheses

The AusCP MSK primary study objective is to:

1. Identify early biomarkers of onset/progression of musculoskeletal complications (hip displacement, scoliosis, fragility fracture) that predict the severity and rate of progression of musculoskeletal complications (MP>30%, Cobb angle>10°, lateral distal femur (LDF) bone mineral density (BMD) Z-score< −2.0) at 8–13 years, leading to natural history data that can be used to inform early intervention clinical trials and identify new therapeutic targets.

The AusCP MSK secondary study objectives are to:

1. Identify the relationship between early brain structure (clinical MRIs), aetiology of CP and musculoskeletal development at 1–4 years of age on disability outcomes at 8–13 years of age.
2. Determine the costs and consequences of medical and allied health resource use (HRU), and poor QoL at 8–13 years of age, related to severity of musculoskeletal complications at 4–9 years of age.
3. Determine prevalence, HRU costs and clinical characteristics of children and adolescents with CP who sustain a pathological fracture, evaluate complementary data for CP patient bone quality and develop a predictive model for fracture.

These aims will be explored through the following hypotheses:

1. Higher prevalence of musculoskeletal risk factors for hip displacement, scoliosis, reduced BMD for age, such as Gross Motor Function Measure (GMFM), HPA/sedentary behaviour, nutritional status, body composition (fat mass/fat free mass on DXA), at 4–9 years of age will lead to poorer musculoskeletal outcomes at 8–13 years of age.
2. The location and extent of the brain lesion(s) on structural MRI, pathophysiology of CP (motor type and distribution), motor capacity (GMFM-88) at 4–9 years of age will predict severity of musculoskeletal complications (hip displacement, scoliosis, bone fragility) and performance outcomes (Pediatric Evaluation of Disability Inventory, Computer Adapted Test, PEDI-CAT) at 8–13 years of age.
3. The location, extent of the brain lesion(s) on semiquantitative MRI (<6 years) will predict the severity of musculoskeletal outcomes (MP>30%, Cobb>10°), at 8–13 years of age.
4. Healthcare costs will be higher for children with greater adverse musculoskeletal biomarkers at 4–9 years of age and will lead to poorer outcomes and QoL (consequences) at 8–13 years of age.
5. Pathologic fracture will be a major health burden in children with CP that increases with GMFCS and age.
6. The identification of children with CP at greatest risk of fracture through a predictive tool will enable the determination of prognosis, inform therapeutic action and reduce fracture burden.
7. Bone quality of children with moderate-to-severe CP will be abnormal compared with TDC;
8. Genetic variation will influence onset and progression of musculoskeletal complications in CP.

### Study design

**Figure 1:**
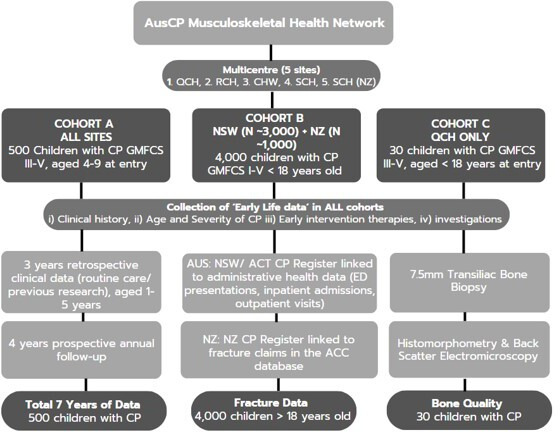
Visual description of the AusCP MSK study cohorts A, B and C. ACC, Accident Compensation Corporation; ACT, Australian Capital Territory; CHW, The Children’s Hospital at Westmead; CP, cerebral palsy; ED, emergency department; GMFCS, Gross Motor Function Classification System; NSW, New South Wales; QCH, Queensland Children’s Hospital; RCH, The Royal Children’s Hospital; SCH (NZ), Starship Children’s Hospital New Zealand.

The AusCP MSK is a multicentre prospective (4 years) and retrospective (3 years) cohort study that will be implemented nationally and internationally at five sites: (1) Queensland Children’s Hospital (QCH); (2) Royal Children’s Hospital, Melbourne (RCH); (3) The Children’s Hospital at Westmead (CHW); (4) The Sydney Children’s Hospital (SCH) and (5) The Starship Children’s Hospital (SCH), New Zealand. This study builds on the foundations of previous studies by the research team: epidemiology of CP, early diagnosis of CP and early surveillance of CP and will undertake further comprehensive surveillance in three cohorts (A, B and C) of children with CP, outlined in figure 1. The study commenced in February 2024 and will be completed by 29 February 2028.

*Cohort A* (n=500) involves the collection of 3-year retrospective data and subsequent 4-year prospective follow-up (baseline (T1) and three annual assessments (T2, T3 and T4)) for a range of musculoskeletal measures with children with CP GMFCS III–V, capturing development from ages 4 to 13 years, providing 7 years of data. This approach enables the identification of early biomarkers of musculoskeletal change, acknowledging that while prospective tracking begins at age 4, important features of musculoskeletal health before this age can be explored through retrospective data collection. *Cohort B* focuses on a retrospective analysis of data from individual’s records from both Australian and New Zealand cohorts to identify clinical presentations for fractures among children with CP GMFCS I–V, aged less than 18 years of age from 2001. The Australian cohort (n~3000) will be retrieved from the NSW/Australian Capital Territory (ACT) CP Register that has been linked with admitted hospital, emergency department and outpatient data collections. The New Zealand cohort (n=~1000) will involve extracting clinical data from the New Zealand Cerebral Palsy Register and linking it to deidentified fracture claims data in the Accident Compensation Corporation database. *Cohort C* includes a single-timepoint examination of bone quality, quantity and turnover in up to 30 children with CP, GMFCS III–V, undergoing scheduled hip surgery at QCH with an exposed iliac crest. A transiliac bone biopsy will be performed using a 5 mm to 7.5 mm diameter trephine. This biopsy will be subjected to testing of histomorphometry and quantitative backscatter electron microscopy imaging (qBEI) for the analysis of: (1) bone cellular activity and (2) BMDD.^82^

### Eligibility criteria

#### Australian sample

Participants are eligible to be included in the prospective studies (cohort A and C) if the following criteria apply: (1) have a diagnosis of CP and gross motor function classified at GMFCS levels III–V; (2) aged between 4.00 and 9.99 years of age at the time of recruitment (any age less than 18.00 years for cohort C); (3) have a parent or legal guardian that is capable of giving signed, informed consent.

Participants will be excluded if the parent/legal guardians’ English language skills are not sufficient to understand the study information, provide informed consent and/or complete the parent-reported study questionnaires.

For study B (cohort B), data extracted from the NSW/ACT CPR will allow records for individuals with CP (GMFCS I–V), aged less than 18 years to be matched in data linkage. The CPR is a population-based database registry with multiple ascertainment strategies of individuals with CP who were born or live in NSW or ACT.

#### New Zealand sample

Participants are eligible to be included in the prospective studies (cohort A) if the following criteria apply: (1) have a diagnosis of CP and gross motor function classified at GMFCS levels III–V; (2) aged between 4.00 and 9.99 years of age at the time of recruitment. For study B (cohort B), data extracted from the New Zealand Cerebral Palsy Register will be linked to fracture claims data in the Accident Compensation Corporation database for children with CP (GMFCS I–V) aged less than 18 years.

##### Outcomes

For cohort groups (A, B and C) the following *retrospective early life data* will be collected at one timepoint only (baseline, T1):

I. Where available, clinical history will be retrieved from medical records, participating registries, clinical trial and neonatal discharge summaries including: sex, ethnicity, gestational age at birth, birth weight, delivery, singleton or one of multiple births, perinatal risk factors (genetic history, infection) and MRI brain findings.

II. Age and severity of CP information will include: age at CP diagnosis, GMFCS,^83^ Manual Ability Classification System (MACS),^84^ Communication Function Classification System (CFCS)^85^ and the Eating and Drinking Assessment Classification System (EDACS).^86^

III. Parent-reported early intensive therapy interventions will include: (1) age at commencement (2), the frequency and duration (dose) and (3) mode (cohort A, C)

IV. Investigations will include: X-rays of hip and/or spine, biochemistry including 25-hydroxy vitamin D (250HD) and alkaline phosphatase (cohort A, C).

Prospective longitudinal study outcomes for cohort A and C, except for DXA scans, will be measured at baseline (T1, year 1), 12 months postbaseline (T2, year 2), 2 years postbaseline (T3, year 3) and 3 years postbaseline (T4, year 4). The AusCP MSK schedule of assessments for all cohort studies (A, B, C) is outlined in table 1.

**Table 1:** Data pooling plan for AusCP MSK studies

Retrospective study cohort A at 1–5 years/cohort B at fracture			Prospective follow-up from 4 to 9 yearsCohorts A and C		
Domain/test	**A**	**B**	TEST	**A**	**C**
**Child primary outcomes**					
1.Hip displacement (MP, AI)	✓		Hip displacement (MP, AI);	✓	✓
Scoliosis: PA/AP and lateral	✓		Scoliosis PA/AP and Lateral	✓	✓
DXA, pQCT			DXA, pQCT	✓	✓
Motor function: GMFM/GMFCS,	✓	✓	Motor function: GMFM/ GMFCS/MACS/CFCS.	✓	✓
Musculoskeletal: ROM assessment (lower limb and spine) including motor type/distribution.	✓		Musculoskeletal: ROM assessment (lower limb and spine) including motor type/distribution	✓	✓
Fractures: hospital admission, emergency department presentation or outpatient visit	✓	✓			
**Child secondary measures are:**					
Function: PEDI-CAT	✓		Function: PEDI-CAT	✓	
Habitual physical activity (5 day)	✓		HPA/sedentary (7 day)	✓	
Growth height/weight/BIA	✓		Growth/growth velocity: Ht/Wt	✓	✓
Skeletal fragility: # history, Pain Q	✓		Skeletal fragility: # history, PPP	✓	✓
Nutrition: 3 day diet, Feeding Q			Feeding Q, Vit D,	✓	✓
Quality of Life—CPQOL	✓		CP CHILD/CHU9	✓	
Mat/neonatal risk	✓	✓	Medical Dx CP, genomics	✓	✓
Neonatal MRI Kiddokoro (aim 2)	✓		Fiori brain lesion severity	✓	
HRU: medical/AH costs	✓	✓	HRU/medical/AH costs/PBS MBS	✓	

AI, Acetabular Index; ANZNN, Australian and New Zealand Neonatal Network; CHU9, Child Health Utility—9 Dimensions; CPCHILD, Caregiver Priorities and Child Health Index of Life with Disabilities; DXA, dual-energy X-ray absorptiometry; GMFM, Gross Motor Function Measure; HEA, Hilgenreiner’s Epiphyseal Angle; HRU, health resource utilisation; MACS, Manual Ability Classification System; MBS, Medicare Benefits Schedule; MP, migration percentage; PBS, Pharmaceutical Benefits Scheme; PEDI_CAT, Paediatric Evaluation of Disability Inventory Computerised Assessment Test; PPP, The Pediatric Pain Profile; pQCT, peripheral Quantitative Computed Tomography.

### Primary outcomes

***Hipassessment*** will involve annual AP pelvis X-rays, performed according to Hip Surveillance Guidelines for positioning children with CP.^29 30^ Reimers MP, acetabular index (AI), sphericity of femoral head, pelvic obliquity, hip abduction/adduction, femoral neck-shaft angle and Hilgenreiner’s epiphysial angle (HEA) will be analysed on all anterior–posterior pelvis X-rays. A passive range of motion (ROM) physical examination will include 12 lower limb assessments performed by the study physiotherapists. The physical examination will measure ROM^87^ of the hip (flexion, extension, abduction, rotation), knee (extension, flexion) and ankle (dorsiflexion), and this measure has demonstrated good interrater,^87 88^ intrarater^88^ and test–retest reliability^89^ in children with CP^87 88^

***Spine assessment*** will use radiological and clinical biomarkers from annual seated anterior–posterior (AP) and lateral spine X-rays (upper thoracic vertebrae to anterior superior iliac spine (ASIS)) to screen and monitor development of scoliosis. Clinical metrics evaluated on spinal X-rays including frontal Cobb angle,^90^ sagittal thoracic kyphosis angle,^90^ coronal sacral shift^91^ and shoulder height asymmetry, sagittal vertical axis,^92^ rotational spinal deformity due to axial rotation,^93^ and coronal rib vertebral angle at the apical vertebra will be utilised as a secondary proxy for rotational deformity.^94^ Clinical assessment of scoliosis will involve the Adam’s forward bend test, and CPUP assessment.^95^ Spinal symmetry will be assessed through a seated lateral flexion test and lateral functional reach test (FRT) and analysed on participants with function at GMFCS III-IV that have been identified as able to complete the test. The seated lateral flexion test involves children in a seated position with arms at their sides using lateral trunk flexion to reach their fingertips down towards the floor on each side. This measure has not been previously psychometrically evaluated in a CP child sample. This study therefore will report on the reliability and predictive validity of this measure for this population. The seated lateral FRT involves children in a seated position with their arm outstretched at a 90° angle and then using their trunk to reach as far across as possible with their arm running parallel with the floor and has high test-retest reliability.^96^ The standing version of this measure has been found to be reliable and valid in ambulant children with CP (GMFCS I – III) compared with TDC^97^ but has not been tested in those at GMFCS levels IV-V.

***Dual energy X-rayabsorptiometry(DXA)*** scans (GE-Lunar (GE Medical Systems) or Hologic [Hologic Inc, USA]) will be acquired at T1 and T3, and at T4 if a low BMD for age (Z-score<−1.5) at any site is reported at T3. Measures will include age and height matched areal BMD (aBMD; g/cm^2^), bone mineral content (BMC; grams) at proximal hip, anteroposterior (AP) lumbar spine, total body and LDF.^98^ These have been demonstrated to be reproducible in CP samples.^99^ Bone mineral apparent density (g/cm^3^) will be used to estimate volumetric BMD from the AP lumbar spine image. The total radiation exposure to provide all DXA scans at three timepoints (T1, T3 and T4) would equate to a total radiation exposure of 0.0075 mSv above normal clinical care. Changes in DXA values will be adequately determined by a baseline (T1), 24-month (T3) and 36 months (T4) assessment. The addition of a 12-month (T2) DXA reading is not expected to significantly enhance the understanding of the natural history of bone mass accrual in this cohort of children.

***TheGMFM*** is a criterion-referenced observation measure developed to measure gross motor function of children with CP.^83^ The GMFM-88 version is used to assess gross motor activities in five dimensions (lying/rolling; sitting; crawling/kneeling, standing; walking, running and jumping).

***Fracture*** sustained for any participants (retrospective and prospective) will be confirmed from patient medical records and using radiological evidence; this will be cross-referenced with an annual parent-reported fracture questionnaire. For cohort B, presentation to emergency department, outpatient clinic or admission to hospital for fracture will be identified using linked administrative health data. Data and age at presentation, procedures and length of stay will be determined.

### Classifications

***Motor type*** will be classified as: spastic, dystonic, ataxic, hypotonic, choreoathetosis, mixed or unclassifiable according to the Surveillance in Europe,^100^ and *spastic topography* as the number of limbs impaired: unilateral (hemiplegia) and bilateral distribution (diplegia, triplegia, quadriplegia) using the Australian Spasticity Assessment Scale.^101^

***Gross Motor Function Classification System (GMFCS)*** has internationally established validity, reliability and stability for the classification of children with CP, 2–12 years.^84^ As gross motor abilities change with age, separate descriptors are used for different age bands using the expanded and revised GMFCS.^102^ This classification system has demonstrated high inter-rater reliability between therapists (ICC=0.84).^103^

***MACS*** is a five-level system developed for children aged 4–18 years with good inter-rater reliability.^84 104^ The MACS classifies how well children use their hands to handle objects in day-to-day activities.^84^ This classification demonstrates high reliability between therapists (ICC=0.97) and between parent and therapist (ICC=0.96).^84^

***CFCS*** has been validated in children aged 2–18 years^85^ and classifies children’s performance in sending and receiving communicative message via their typical communication means. Good inter-rater reliability between professionals has been demonstrated for this measure (κ_w_=0.77) for children older than 4 years and high test–retest reliability 0.82.^85^

***EDACS*** classifies the eating and drinking abilities of children with CP from 3 years of age and describes the level of safety and efficiency (levels I–V) on consuming food and fluid textures.^105^ This classification demonstrates high inter-rater reliability between professionals (ICC=0.93).^86^

### Secondary outcomes

***Peripheral quantitative computer tomography (pQCT)*** of the tibia will be used to measure the volumetric BMD (mg/cm^3^), shape and size of cortical and trabecular bone, and to derive strength measures.^106^ pQCT will be performed on children GMFCS III and subset of GMFCS IV and V, from cohort A. The total radiation dose for the pQCT scans (maximum total of 3 during the study) is 0.03 mSv above normal clinical care.

***Bone biopsy (cohort C)*** will involve a transiliac bone biopsy after tetracycline labelling in 30 subjects with CP, GMFCS III-V who are undergoing scheduled hip surgery. A biopsy from the iliac crest using a 5 mm or 7.5 mm diameter trephine. Bone cellular activity and BMDD on the sample will be measured using qBEI. Measurements will be compared with normative data of BMDD established in TDC.^107^

***HPA*** will be measured using accelerometers worn on the less-affected wrist (ActiGraph GT3X+) and less-affected thigh (Axivity AX3) for 7 consecutive days immediately after each assessment visit, where day 1 is defined as the first full day after receipt of the accelerometers. Parents/caregivers will record the child’s sleep times and removal of the devices on an activity log sheet. The raw accelerometer signal will be processed into HPA outcomes using machine-learnt physical activity classification models specifically trained and validated for youth with CP who are ambulatory or use mobility aids for ambulation.^108 109^

***Brain structure*** measured via retrospective structural brain MRIs captured at any age will be assessed by study investigators at Commonwealth Scientific and Industrial Research Organisation using automated, quantitative methods, namely the developing Human Connectome Project structural pipeline^110^ or neonatal data, and the AssessCP for children aged 4–18 years.^111^ This will provide measures of brain macrostructure (regional volumes) and cortical shape (cortical thickness, sulcal depth). This data will also be assessed manually using the Fiori brain lesion severity scale,^112^ cortical thickness, sulcal depth) and to describe brain lesion characteristics. This data will be combined with clinical information to predict GMFM and musculoskeletal complications.

### Growth and nutritional status

1. Anthropometric measures will be performed including height, length, knee height and body weight. Body mass index (BMI) will be calculated. Nutritional status will be determined using Z-score cut-offs for height, weight and BMI for each participant, relative to Centres for Disease Control and Prevention 2000 growth data.^113^
2. Body composition: DXA measurements of fat mass, lean body mass and BMC.^114^
3. Feeding behaviours will be assessed through a parent report questionnaire,^113^ the Feeding Nutrition Screening Tool,^115^ the Functional Oral Intake Scale^116^ and the Australian Recommended Food Score.^117^
4. Vitamin D and calcium intake will be collected via a parent-reported food frequency questionnaire, with consideration given to supplementation.^118^

***Pain*** variables including episodes of pain, treatment and medication will be collected using the parent-reported Paediatric Pain Profile (PPP).^119^ The PPP rates severity of pain ‘at best’ and ‘while in pain’ with good internal consistency (α=0.75–0.89) and reliability (ICC 0.74–0.89).^120^ In instances where participants can self-report pain, the Wong-Baker Faces Pain Scale will be used.^120^

***Sexual maturation*** will be measured through parent-reported pubertal status of the child via pictorial illustrations, where puberty will be defined by Tanner stage ≥2.^121^ Children with delayed or precocious puberty will have their clinical status highlighted to their treating medical team.

***Biochemistry*** will involve blood collected at each timepoint for markers of optimal growth and mineral homeostasis, including 25-hydroxy vitamin D (25OHD), calcium and alkaline phosphatase, which are altered in CP related to GMFCS, body composition, growth and nutrition.^122^

***Sun exposure***will be measured through a parent-reported 7-day sun exposure diary within 2 weeks of the 25OHD levels to record the child’s daily UV exposure,^123^ concurrent with the 7-day physical activity monitoring.

***Genetic testing*** will involve DNA from children in cohort A that will be biobanked for future use to identify potential pathogenic variants in genes implicated in CP and comorbid conditions including musculoskeletal complications. This is an optional substudy. Individuals who do not wish to participate in the genetic research may still participate in the main study. A 4 mL EDTA blood sample for DNA isolation will be collected from participants who have consented to this substudy.

### Parent-reported secondary outcomes

The following parent-reported outcome measures will be completed by the child’s parent/guardian or caregiver(s) and assisted by the assessor at each timepoint:

***The Child Health Utility-9 Dimensions*****(CHU9D)** provides a preference-based utility index for assessing health-related QoL (HRQL) in children where the nine dimensions reflect paediatric perspectives of HRQL.^124^ The parent proxy version will be used, completed by the parent or guardian to provide a utility index for economic evaluations.^125^ The CHU9D has demonstrated moderate test–retest reliability (ICC 0.52 and 0.60).^126^

***The Caregiver Priorities andChildHealth Index of Life with Disabilities (CPCHILD)*****^127^**questionnaire assesses HRQoL across six domains: Personal Care and Activities of Daily Living; Positioning, Transferring and Mobility; Comfort/Emotions; Communication & Social Interaction; Health and Overall QOL. The CPCHILD has demonstrated content validity, reliability and construct validity for ease of caregiving, functional limitations, comfort, health and well-being of children with severe CP, test–retest reliability (ICC 0.97 and 0.88–0.96 for domain scores) and internal consistency (Cronbach’s alpha>0.70).^127^

***The Pediatric Evaluation of Disability Inventory Computer Adaptive Test (PEDI-CAT)*** is a valid and reliable parent-reported assessment of a child’s function in four domains^128^: Daily activities; Mobility; Social/Cognitive and Responsibility, using scaled scores (Rasch) that have good validity and reliability.^129^ Scaled scores (possible range 20–80) for each domain provide more precise results in the extreme ranges, compared with normative standard scores, and are recommended to track function progress in children with substantial delays.^129^

***Health Resource Use (HRU)*** questionnaire^130^ will be completed by the parent or guardian and supplemented by Medicare Benefits Schedule (MBS) and Pharmaceutical Benefits Scheme (PBS) data. The HRU includes data on screening, therapy frequency and duration, hospital admissions, medical visits, operations, medication, equipment and parent time for appointments. Standard cost sources (MBS, PBS, hospital, diagnosis-related groups for inpatient and outpatient services) will be applied to resource use and total cost of care calculated per child aged 2–12 years. The cost–consequence analysis will report health outcomes to demonstrate value for money, including best possible musculoskeletal health, physical function, greater QoL, non-health effects and non-patient effects (carer well-being and QoL).

### Sample size and power calculations

The total number of participants with data in line with the primary study objectives is expected to be n=450. This assumes 4 years of data from 90% of 500 enrolled participants and is based on attrition rates in previous National Health and Medical Research Council (NHMRC) funded cohorts run by the CI team. If the HD prevalence in approximately 70% of the total cohort is assumed, the AusCP MSK study will have 90% statistical power (α=0.05) to detect absolute differences between binary risk factor categories exceeding 12%. This corresponds to detecting relative risks (RRs) falling below 0.80 or surpassing 1.24. Even when risk factors are unbalanced in a 4:1 ratio, we will be able to identify RRs of <0.74 or >1.35. For scoliosis incidence, if an overall cumulative incidence of 25% is assumed, the study will be able to detect absolute between-group differences of >11%. Note that this is an underestimate of power due to the repeated annual measurements for each child.

The bone quality cohort (C) will recruit 30 children with CP, detecting between-group differences >1.25 SD with 90% power, which are smaller differences than observed when comparing children with mild Osteogenesis Imperfecta compared with TDC.^131^

#### Statistical analysis

**Figure 2:**
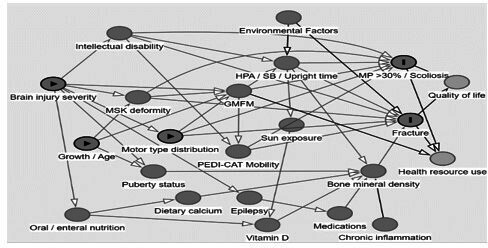
Directed acyclic graph model of musculoskeletal complications in children with cerebral palsy. GMFM, Gross Motor Function Measure; HPA, habitual physical activity; MP, migration percentage; PEDI-CAT, Pediatric Evaluation of Disability Inventory, Computer Adapted Test; SB, sedentary behaviour.

Data will be stored on the research data manager hosted by lead university (The University of Queensland). To identify relationships between early biomarkers (aim 1), early brain structure (aim 2) and clinical characteristics and bone quality (aim 4) with musculoskeletal complications, we will construct multivariable regression models informed by the directed acyclic graph displayed in figure 2. The association between risk factors and primary outcomes measured on the interval scale (ie, MP%) will be investigated using mixed-effects linear regression models with child included as a random effect to account for repeated measures. Risk factor and time will be included as fixed effects, with an interaction term. Potential confounding variables will be included. For binary outcomes, mixed effects logistic regression models will be constructed and mixed effects Poisson models used for count outcomes.

When constructing predictive models, important variables will be examined individually first before a final multivariable model is selected using Bayesian information criteria. Model calibration will be tested graphically, and internal calibration will use bootstrap resampling. Missing data will be treated as case-by-case basis depending on the observed pattern of missingness. For example, if data are ‘missing at random’, we will use multiple imputation methods and if data are ‘not missing at random’, we will use a pattern-mixture model. Adjustments for multiple comparisons will be made for each separate suite of analyses as appropriate, bearing in mind the type I and type II error rates for each suite.

In addition to the primary statistical analysis, a cost–consequence analysis will be performed to attain a better understanding of the value and costs of a national approach to better decision-making regarding hip, spine and bone fragility complications in CP. The cost–consequence analysis will capture the direct costs of medical care and direct non-medical costs from the HRU parent-reported questionnaire. The cost-consequence analysis will report costs and outcomes in a simple and disaggregated format to help policymakers better understand the effects of prevention programmes for musculoskeletal problems across costs and outcomes. The analysis will be performed in a 4-year time horizon with a 3% discount rate to account for time preference.

## Ethics and **dissemination**

The AusCP MSK Health Network has been approved by the Children’s Health Queensland Hospital and Health Service Human Research Ethics Committee (HREC/22/QCHQ87118), The University of Queensland Human Research Ethics Committee (2022/ HE001516) and the New Zealand Health and Disability Ethics Committee (2025/FULL/21456). The trial is also registered on the Australian New Zealand Clinical Trials Registry (ANZCTR, ACTRN12622000782730p). The trial registration will be amended to reflect any protocol updates, and deviations from the protocol will be reported in the primary results manuscript. Study outcomes will be disseminated via the ANZCTR website, conference presentations and abstracts, peer-reviewed scientific journals, organisation/institution media releases and newsletters and directly to research participants in an appropriate and accessible format.

### Patient and public Involvement

An AusCP MSK consumer group, consisting of parents/families/caregivers with lived experience, has been established to provide ongoing guidance and input throughout the research process. Consumer representatives will be involved at all research stages, including study design, creation of parent/participant information and consent materials (see onlinesupplemental files 1^3^), selection of assessment materials and procedures, study implementation and dissemination of results. Ongoing broader consumer engagement will continue via the consumer network (Aus-CP-CTN CRE). The AusCP MSK consumer representatives will be financially compensated for their time and expertise. Consumer representatives will nominate their preferred method of payment (either direct credit into their account or gift card). The rate of reimbursement will be equivalent to Health Consumer Queensland remuneration rates, adjusted for annual inflation increases (1.5%). Consumer council meetings will be held no less than two times yearly, and one consumer representative will be invited to sit on the trial management committee.

## Supplementary material

10.1136/bmjopen-2024-095526 online supplemental file 1

10.1136/bmjopen-2024-095526 online supplemental file 2

10.1136/bmjopen-2024-095526 online supplemental file 3

## References

[1] ACPR (2023). Report of the australian cerebral palsy register, birth years 1995-2016.

[2] Bax M, Goldstein M, Rosenbaum P (2005). Proposed definition and classification of cerebral palsy, April 2005. Dev Med Child Neurol.

[3] Rosenbaum P, Paneth N, Leviton A (2007). A report: the definition and classification of cerebral palsy April 2006. Developmental Medicine & Child Neurology.

[4] Chen W, Khan Z, Freund J (2022). Dual Hip DXA. Is it Time to Change Standard Protocol?. J Clin Densitom.

[5] Gordon CM, Zemel BS, Wren TAL (2017). The Determinants of Peak Bone Mass. J Pediatr.

[6] Jepsen KJ, Silva MJ, Vashishth D (2015). Establishing biomechanical mechanisms in mouse models: practical guidelines for systematically evaluating phenotypic changes in the diaphyses of long bones. J Bone Miner Res.

[7] Howard JJ, Graham K, Shortland AP (2022). Understanding skeletal muscle in cerebral palsy: a path to personalized medicine?. Develop Med Child Neuro.

[8] Bache CE, Selber P, Graham HK (2003). The management of spastic diplegia. Curr Orthop.

[9] Ward KD, Chiarello LA, Bartlett DJ (2014). Ease of Caregiving for Children: a measure of parent perceptions of the physical demands of caregiving for young children with cerebral palsy. Res Dev Disabil.

[10] Houlihan CM, O’Donnell M, Conaway M (2004). Bodily pain and health-related quality of life in children with cerebral palsy. Dev Med Child Neurol.

[11] Barber LEE, Hastings-ison T, Baker R (2011). Medial gastrocnemius muscle volume and fascicle length in children aged 2 to 5 years with cerebral palsy. Developmental Medicine & Child Neurology.

[12] Graham HK (2001). Botulinum toxin type A management of spasticity in the context of orthopaedic surgery for children with spastic cerebral palsy. Euro J of Neurology.

[13] Opheim A, Jahnsen R, Olsson E (2012). Balance in relation to walking deterioration in adults with spastic bilateral cerebral palsy. Phys Ther.

[14] Skoutelis VC, Kanellopoulos AD, Kontogeorgakos VA (2020). The orthopaedic aspect of spastic cerebral palsy. J Orthop.

[15] Barrett RS, Lichtwark GA (2010). Gross muscle morphology and structure in spastic cerebral palsy: a systematic review. Develop Med Child Neuro.

[16] Aroojis A, Mantri N, Johari AN (2021). Hip Displacement in Cerebral Palsy: The Role of Surveillance. Indian J Orthop.

[17] Dohin B (2019). The spastic hip in children and adolescents. Orthopaedics & Traumatology: Surgery & Research.

[18] Al-Rumaih MH, Camp MW, Narayanan UG (2023). Current Concept and Management of Spastic Hip in Children: A Narrative Review. Cureus.

[19] Larkin-Kaiser KA, Howard JJ, Leonard T (2019). Relationship of muscle morphology to hip displacement in cerebral palsy: a pilot study investigating changes intrinsic to the sarcomere. J Orthop Surg Res.

[20] Hasler C, Brunner R, Grundshtein A (2020). Spine deformities in patients with cerebral palsy; the role of the pelvis. J Child Orthop.

[21] Presedo A, Dabney KW, Miller F (2007). Fractures in patients with cerebral palsy. J Pediatr Orthop.

[22] Palisano R, Rosenbaum P, Walter S (1997). Development and reliability of a system to classify gross motor function in children with cerebral palsy. Develop Med Child Neuro.

[23] Hägglund G, Lauge-Pedersen H, Wagner P (2007). Characteristics of children with hip displacement in cerebral palsy. BMC Musculoskelet Disord.

[24] Soo B, Howard JJ, Boyd RN (2006). Hip displacement in cerebral palsy. J Bone Joint Surg Am.

[25] Robin J, Graham HK, Selber P Proximal femoral geometry in cerebral palsy: a population-based cross-sectional study. The Journal of Bone and Joint Surgery British.

[26] Ramstad K, Jahnsen RB, Terjesen T (2017). Severe hip displacement reduces health-related quality of life in children with cerebral palsy. Acta Orthop.

[27] Ramstad K, Terjesen T (2016). Hip pain is more frequent in severe hip displacement: a population-based study of 77 children with cerebral palsy. J Pediatr Orthop B.

[28] Gibson N, Wynter M, Thomason P (2022). Australian hip surveillance guidelines at 10 years: New evidence and implementation. J Pediatr Rehabil Med.

[29] Wynter M, Gibson N, Kentish M (2011). The Consensus Statement on Hip Surveillance for Children with Cerebral Palsy: Australian Standards of Care. J Pediatr Rehabil Med.

[30] Wynter M, Gibson N, Willoughby KL (2015). Australian hip surveillance guidelines for children with cerebral palsy: 5‐year review. Develop Med Child Neuro.

[31] Hägglund G, Alriksson-Schmidt A, Lauge-Pedersen H (2014). Prevention of dislocation of the hip in children with cerebral palsy: 20-year results of a population-based prevention programme. Bone Joint J.

[32] Kentish M, Wynter M, Snape N (2011). Five-year outcome of state-wide hip surveillance of children and adolescents with cerebral palsy. J Pediatr Rehabil Med.

[33] Hagglund G, Andersson S, Duppe H (2005). Prevention of dislocation of the hip in children with cerebral palsy. The first ten years of a population-based prevention programme. The Journal of Bone & Joint Journal.

[34] Miller SD, Juricic M, Hesketh K (2017). Prevention of hip displacement in children with cerebral palsy: a systematic review. Develop Med Child Neuro.

[35] Krach LE, Kriel RL, Gilmartin RC (2004). Hip status in cerebral palsy after one year of continuous intrathecal baclofen infusion. Pediatr Neurol.

[36] Floeter N, Lebek S, Bakir MS (2014). Changes in hip geometry after selective dorsal rhizotomy in children with cerebral palsy. Hip Int.

[37] Heim RC, Park TS, Vogler GP (1995). Changes in hip migration after selective dorsal rhizotomy for spastic quadriplegia in cerebral palsy. J Neurosurg.

[38] Hicdonmez T, Steinbok P, Beauchamp R (2005). Hip joint subluxation after selective dorsal rhizotomy for spastic cerebral palsy. Journal of Neurosurgery: Pediatrics.

[39] DiFazio R, Shore B, Vessey JA (2016). Effect of Hip Reconstructive Surgery on Health-Related Quality of Life of Non-Ambulatory Children with Cerebral Palsy. J Bone Joint Surg Am.

[40] Pons C, Rémy‐Néris O, Médée B (2013). Validity and reliability of radiological methods to assess proximal hip geometry in children with cerebral palsy: a systematic review. Develop Med Child Neuro.

[41] Bagg MR, Farber J, Miller F (1993). Long-term follow-up of hip subluxation in cerebral palsy patients. J Pediatr Orthop.

[42] Herndon WA, Bolano L, Sullivan JA (1992). Hip stabilization in severely involved cerebral palsy patients. J Pediatr Orthop.

[43] Zarrinkalam R, Rice J, Brook P (2011). Hip displacement and overall function in severe cerebral palsy. J Pediatr Rehabil Med.

[44] Krebs A, Strobl WM, Grill F (2008). Neurogenic hip dislocation in cerebral palsy: quality of life and results after hip reconstruction. J Child Orthop.

[45] Miller F, Girardi H, Lipton G (1997). Reconstruction of the dysplastic spastic hip with peri-ilial pelvic and femoral osteotomy followed by immediate mobilization. J Pediatr Orthop.

[46] Rutz E, Vavken P, Camathias C (2015). Long-term results and outcome predictors in one-stage hip reconstruction in children with cerebral palsy. J Bone Joint Surg Am.

[47] Vitale MG, Matsumoto H, Bye MR (2008). A retrospective cohort study of pulmonary function, radiographic measures, and quality of life in children with congenital scoliosis: an evaluation of patient outcomes after early spinal fusion. Spine (Phila Pa 1976).

[48] Saito N, Ebara S, Ohotsuka K (1998). Natural history of scoliosis in spastic cerebral palsy. Lancet.

[49] Loeters MJB, Maathuis CGB, Hadders‐algra M (2010). Risk factors for emergence and progression of scoliosis in children with severe cerebral palsy: a systematic review. Develop Med Child Neuro.

[50] Hägglund G, Pettersson K, Czuba T (2018). Incidence of scoliosis in cerebral palsy. Acta Orthop.

[51] Persson-Bunke M, Hägglund G, Lauge-Pedersen H (2012). Scoliosis in a Total Population of Children With Cerebral Palsy. Spine (Phila Pa 1986).

[52] Willoughby KL, Ang SG, Thomason P (2022). Epidemiology of scoliosis in cerebral palsy: A population-based study at skeletal maturity. J Paediatr Child Health.

[53] Ágústsson A, Sveinsson Þ, Rodby-Bousquet E (2017). The effect of asymmetrical limited hip flexion on seating posture, scoliosis and windswept hip distortion. Res Dev Disabil.

[54] Casey J, Agustsson A, Rosenblad A (2022). Relationship between scoliosis, windswept hips and contractures with pain and asymmetries in sitting and supine in 2450 children with cerebral palsy. Disabil Rehabil.

[55] Hägglund G (2020). Association between pelvic obliquity and scoliosis, hip displacement and asymmetric hip abduction in children with cerebral palsy: a cross-sectional registry study. BMC Musculoskelet Disord.

[56] Cloake T, Gardner A (2016). The management of scoliosis in children with cerebral palsy: a review. J Spine Surg.

[57] Terjesen T, Lange JE, Steen H (2000). Treatment of scoliosis with spinal bracing in quadriplegic cerebral palsy. Develop Med Child Neuro.

[58] Miller A, Temple T, Miller F (1996). Impact of orthoses on the rate of scoliosis progression in children with cerebral palsy. J Pediatr Orthop.

[59] Olafsson Y, Saraste H, Al-Dabbagh Z (1999). Brace treatment in neuromuscular spine deformity. J Pediatr Orthop.

[60] Angsupaisal M, Maathuis CGB, Hadders‐Algra M (2015). Adaptive seating systems in children with severe cerebral palsy across International Classification of Functioning, Disability and Health for Children and Youth version domains: a systematic review. Develop Med Child Neuro.

[61] Henderson RC, Kairalla J, Abbas A (2004). Predicting low bone density in children and young adults with quadriplegic cerebral palsy. Develop Med Child Neuro.

[62] Houlihan C, Kuperminc M, Gurka M (2009). Longitudinal assessment of low bone mineral density and its association with severe pain in children with cerebral palsy. Developmental Medicine & Child Neurology.

[63] Whitney DG, Caird MS, Jepsen KJ (2022). Excess healthcare spending associated with fractures among adults with cerebral palsy. Disabil Health J.

[64] Henderson RC, Grossberg RI, Matuszewski J (2007). Growth and nutritional status in residential center versus home-living children and adolescents with quadriplegic cerebral palsy. J Pediatr.

[65] Kannikeswaran S, French ZP, Walsh K (2022). Fracture characteristics by age, sex, and ambulatory status among individuals with cerebral palsy: a descriptive study. Disabil Rehabil.

[66] Stevenson RD, Conaway M, Barrington JW (2006). Fracture rate in children with cerebral palsy. Pediatr Rehabil.

[67] Linton G, Hägglund G, Czuba T (2022). Epidemiology of fractures in children with cerebral palsy: a Swedish population-based registry study. BMC Musculoskelet Disord.

[68] Damiano DL (2006). Activity, activity, activity: rethinking our physical therapy approach to cerebral palsy. Phys Ther.

[69] Daci E, van Cromphaut S, Bouillon R (2002). Mechanisms influencing bone metabolism in chronic illness. Horm Res.

[70] Munns CF, Cowell CT (2005). Prevention and treatment of osteoporosis in chronically ill children. Journal of Musculoskeletal and Neuronal Interactations.

[71] Simm PJ, Biggin A, Zacharin MR (2018). Consensus guidelines on the use of bisphosphonate therapy in children and adolescents. J Paediatr Child Health.

[72] Hurley T, Zareen Z, Stewart P (2021). Bisphosphonate use in children with cerebral palsy. Cochrane Database Syst Rev.

[73] Ozel S, Switzer L, Macintosh A (2016). Informing evidence‐based clinical practice guidelines for children with cerebral palsy at risk of osteoporosis: an update. Develop Med Child Neuro.

[74] Nguyen ND, Frost SA, Center JR (2007). Development of a nomogram for individualizing hip fracture risk in men and women. Osteoporos Int.

[75] Nguyen ND, Frost SA, Center JR (2008). Development of prognostic nomograms for individualizing 5-year and 10-year fracture risks. Osteoporos Int.

[76] Liquori BM, Gannotti ME, Thorpe DE (2022). Characteristics of Interventions to Improve Bone Health in Children With Cerebral Palsy: A Systematic Review. Pediatr Phys Ther.

[77] Chad KE, Bailey DA, McKay HA (1999). The effect of a weight-bearing physical activity program on bone mineral content and estimated volumetric density in children with spastic cerebral palsy. J Pediatr.

[78] Wren TAL, Lee DC, Hara R (2010). Effect of high-frequency, low-magnitude vibration on bone and muscle in children with cerebral palsy. J Pediatr Orthop.

[79] Novak I, Morgan C, Adde L (2017). Early, Accurate Diagnosis and Early Intervention in Cerebral Palsy: Advances in Diagnosis and Treatment. JAMA Pediatr.

[80] Morgan C, Fetters L, Adde L (2021). Early Intervention for Children Aged 0 to 2 Years With or at High Risk of Cerebral Palsy: International Clinical Practice Guideline Based on Systematic Reviews. JAMA Pediatr.

[81] Jackman M, Sakzewski L, Morgan C (2022). Interventions to improve physical function for children and young people with cerebral palsy: international clinical practice guideline. Develop Med Child Neuro.

[82] Castoldi NM, Pickering E, Sansalone V (2024). Bone turnover and mineralisation kinetics control trabecular BMDD and apparent bone density: insights from a discrete statistical bone remodelling model. Biomech Model Mechanobiol.

[83] Palisano RJ, Hanna SE, Rosenbaum PL (2000). Validation of a model of gross motor function for children with cerebral palsy. Phys Ther.

[84] Eliasson A-C, Krumlinde-Sundholm L, Rösblad B (2006). The Manual Ability Classification System (MACS) for children with cerebral palsy: scale development and evidence of validity and reliability. Dev Med Child Neurol.

[85] Hidecker MJC, Paneth N, Rosenbaum PL (2011). Developing and validating the Communication Function Classification System for individuals with cerebral palsy. Dev Med Child Neurol.

[86] Sellers D, Mandy A, Pennington L (2014). Development and reliability of a system to classify the eating and drinking ability of people with cerebral palsy. Develop Med Child Neuro.

[87] Fosang AL, Galea MP, McCoy AT (2003). Measures of muscle and joint performance in the lower limb of children with cerebral palsy. Dev Med Child Neurol.

[88] McWhirk LB, Glanzman AM (2006). Within-session inter-rater realiability of goniometric measures in patients with spastic cerebral palsy. Pediatr Phys Ther.

[89] Klingels K, De Cock P, Molenaers G (2010). Upper limb motor and sensory impairments in children with hemiplegic cerebral palsy. Can they be measured reliably?. Disabil Rehabil.

[90] Cobb JR (1948). Outlines for the study of scoliosis. Journal of Bone and Joint Surgery.

[91] Thawrani D, Agabegi SS, Eismann E (2013). Accuracy and Reliability of Drawing Central Sacral Vertical Line on Scoliosis Radiographs in Clinical Practice. Spine Deform.

[92] Diebo BG, Varghese JJ, Lafage R (2015). Sagittal alignment of the spine: What do you need to know?. Clin Neurol Neurosurg.

[93] Nash CL, Moe JH (1969). A Study of Vertebral Rotation. The Journal of Bone & Joint Surgery.

[94] Mehta MH (1972). The rib-vertebra angle in the early diagnosis between resolving and progressive infantile scoliosis. J Bone Joint Surg Br.

[95] Persson-Bunke M, Czuba T, Hägglund G (2015). Psychometric evaluation of spinal assessment methods to screen for scoliosis in children and adolescents with cerebral palsy. BMC Musculoskelet Disord.

[96] Park S-H, Son S-M, Choi J-Y (2021). Effect of posture control training using virtual reality program on sitting balance and trunk stability in children with cerebral palsy. NeuroRehabilitation.

[97] Dewar RM, Tucker K, Claus AP (2021). Postural Control Performance on the Functional Reach Test: Validity of the Kids-Balance Evaluation Systems Test (Kids-BESTest) Criteria. Arch Phys Med Rehabil.

[98] Henderson RC, Berglund LM, May R (2010). The relationship between fractures and DXA measures of BMD in the distal femur of children and adolescents with cerebral palsy or muscular dystrophy. J Bone Miner Res.

[99] Zemel BS, Stallings VA, Leonard MB (2009). Revised pediatric reference data for the lateral distal femur measured by Hologic Discovery/Delphi dual-energy X-ray absorptiometry. J Clin Densitom.

[100] Surveillance of Cerebral Palsy in Europe (SCPE) (2000). Surveillance of cerebral palsy in Europe: a collaboration of cerebral palsy surveys and registers. Dev Med Child Neurol.

[101] Love S, Gibson N, Smith N (2016). Interobserver reliability of the Australian Spasticity Assessment Scale (ASAS). Develop Med Child Neuro.

[102] Palisano RJ, Rosenbaum P, Bartlett D (2008). Content validity of the expanded and revised Gross Motor Function Classification System. Develop Med Child Neuro.

[103] Bodkin AW, Robinson C, Perales FP (2003). Reliability and validity of the gross motor function classification system for cerebral palsy. Pediatr Phys Ther.

[104] Morris C, Kurinczuk JJ, Fitzpatrick R (2006). Reliability of the manual ability classification system for children with cerebral palsy. Dev Med Child Neurol.

[105] Sellers D, Bryant E, Hunter A (2019). The Eating and Drinking Ability Classification System for cerebral palsy: A study of reliability and stability over time. J Pediatr Rehabil Med.

[106] Rauch F, Land C, Cornibert S (2005). High and low density in the same bone: a study on children and adolescents with mild osteogenesis imperfecta. Bone.

[107] Fratzl-Zelman N, Roschger P, Misof BM (2009). Normative data on mineralization density distribution in iliac bone biopsies of children, adolescents and young adults. Bone.

[108] Ahmadi M, O’Neil M, Fragala-Pinkham M (2018). Machine learning algorithms for activity recognition in ambulant children and adolescents with cerebral palsy. J Neuroeng Rehabil.

[109] Goodlich BI, Armstrong EL, Horan SA (2020). Machine learning to quantify habitual physical activity in children with cerebral palsy. Develop Med Child Neuro.

[110] Makropoulos A, Robinson EC, Schuh A (2018). The developing human connectome project: A minimal processing pipeline for neonatal cortical surface reconstruction. Neuroimage.

[111] Pagnozzi AM, Dowson N, Doecke J (2017). Identifying relevant biomarkers of brain injury from structural MRI: Validation using automated approaches in children with unilateral cerebral palsy. PLoS ONE.

[112] Fiori S, Cioni G, Klingels K (2014). Reliability of a novel, semi‐quantitative scale for classification of structural brain magnetic resonance imaging in children with cerebral palsy. Develop Med Child Neuro.

[113] Oftedal S, Davies PSW, Boyd RN (2016). Longitudinal Growth, Diet, and Physical Activity in Young Children With Cerebral Palsy. Pediatrics.

[114] Blue MNM, Hirsch KR, Brewer GJ (2022). The validation of contemporary body composition methods in various races and ethnicities. Br J Nutr.

[115] Bell KL, Benfer KA, Ware RS (2019). Development and validation of a screening tool for feeding/swallowing difficulties and undernutrition in children with cerebral palsy. Dev Med Child Neurol.

[116] Yi YG, Shin HI (2020). Psychometrics of the Functional Oral Intake Scale for Children With Dysphagia. J Pediatr Gastroenterol Nutr.

[117] Andrisevic E, Westberry DE, Pugh LI (2016). Correction of Tibial Torsion in Children With Cerebral Palsy by Isolated Distal Tibia Rotation Osteotomy: A Short-term, In Vivo Anatomic Study. J Pediatr Orthop.

[118] Taylor C, Lamparello B, Kruczek K (2009). Validation of a food frequency questionnaire for determining calcium and vitamin D intake by adolescent girls with anorexia nervosa. J Am Diet Assoc.

[119] Hunt A, Goldman A, Seers K (2004). Clinical validation of the Paediatric Pain Profile. Develop Med Child Neuro.

[120] Garra G, Singer AJ, Taira BR (2010). Validation of the Wong-Baker FACES Pain Rating Scale in pediatric emergency department patients. Acad Emerg Med.

[121] Tanner JM (1962). Growth at Adolescence: With a General Consideration of the Effects of Hereditary and Environmental Factors Upon Growth and Maturation from Birth to Maturity.

[122] Munns C, Zacharin MR, Rodda CP (2006). Prevention and treatment of infant and childhood vitamin D deficiency in Australia and New Zealand: a consensus statement. Med J Aust.

[123] Harrison SL, Buettner PG, Maclennan R (2005). The North Queensland “Sun-Safe Clothing” study: design and baseline results of a randomized trial to determine the effectiveness of sun-protective clothing in preventing melanocytic nevi. Am J Epidemiol.

[124] Stevens K, Ratcliffe J (2012). Measuring and valuing health benefits for economic evaluation in adolescence: an assessment of the practicality and validity of the child health utility 9D in the Australian adolescent population. Value Health.

[125] Furber G, Segal L (2015). The validity of the Child Health Utility instrument (CHU9D) as a routine outcome measure for use in child and adolescent mental health services. Health Qual Life Outcomes.

[126] Xiong X, Carvalho N, Huang L (2024). (PedsQL). Pharmacoeconomics.

[127] Narayanan UG, Fehlings D, Weir S (2006). Initial development and validation of the Caregiver Priorities and Child Health Index of Life with Disabilities (CPCHILD). Dev Med Child Neurol.

[128] Shore BJ, Allar BG, Miller PE (2019). Measuring the Reliability and Construct Validity of the Pediatric Evaluation of Disability Inventory-Computer Adaptive Test (PEDI-CAT) in Children With Cerebral Palsy. Arch Phys Med Rehabil.

[129] Haley SM, New England Medical Center H, Group PR (1992). Pediatric Evaluation of Disability Inventory (PEDI). Development, Standardization and Administration Manual.

[130] Boyd RN, Jordan R, Pareezer L (2013). Australian Cerebral Palsy Child Study: protocol of a prospective population based study of motor and brain development of preschool aged children with cerebral palsy. BMC Neurol.

[131] Roschger P, Fratzl-Zelman N, Misof BM (2008). Evidence that abnormal high bone mineralization in growing children with osteogenesis imperfecta is not associated with specific collagen mutations. Calcif Tissue Int.

