# Supplementary material for: Study protocol for the Australasian Cerebral Palsy Musculoskeletal Health Network (AusCP MSK) prospective cohort study: early detection of musculoskeletal complications in young children with moderate to severe cerebral palsy (GMFCS III–V)
Source: BMJ Open. 2025 Apr 30;15(4):e095526. doi: 10.1136/bmjopen-2024-095526 (PMC12049940; doi:10.1136/bmjopen-2024-095526)
Supplement: online supplemental file 1 [file bmjopen-15-4-s001.pdf]

Parent/Guardian Information and Consent Form

**Title**	The Australian Cerebral Palsy Musculoskeletal Health Network Study (AusCP MSK)
**Protocol Number**	HREC/2022/QCHQ/87118
**Project Sponsor (if applicable)**	The University of Queensland
**Principal Investigator (PI)**	*Insert Site PI here*
**Location (where PI will recruit)**	*Insert Site Here*

Thank you for taking the time to read this Parent/Guardian Information Statement and Consent Form. We would like to ask your child to participate in a research project that is explained below.

It is ok to say no

What is an Information Statement?

These pages tell you about the research project. It explains to you clearly and openly all the steps and procedures of the project. The information is to help you decide whether or not you would like your child to take part in the research. Please read this Information Statement carefully.

Before you decide if you want your child to take part or not, you can ask us any questions you have about the project. You may want to talk about the project with your family, friends, or health care worker.

Important things to know

- It is your choice whether or not your child can take part in the research. You do not have to agree if you do not want to.
- If you decide you do not want your child to take part, it will not affect the treatment and care your child receives through Children's Health Queensland

If you decide you want your child to take part in the research project, you will be asked to sign the consent section. By signing it you are telling us that you:

- Understand what you have read
- Consent to the child taking part in the research project
- Consent for the child to have the tests and treatments that are described
- Consent to the use of the child's personal and health information as described.

You will be given a copy of this Participant Information and Consent Form to keep.

1. What is genetic research?

Genetic research is important to advance science and public health.

This optional research project will require the analysis of your child's genetic information. You can think of this genetic information as a large instruction book that your body reads to understand how it should be built and function. All humans have the same instruction book in their body, but some words or letters may be different from one person to the other. Some of those differences have no effect on your health but others can influence the likelihood of developing a disease or affect how medicines to treat a disease will work. These genetic analyses may involve looking at part or all of your child's genetic information.

The main AusCP MSK study is looking at your child's musculoskeletal health as they grow. This optional sub-study will test your child's DNA to see if we can identify if there is a genetic component to musculoskeletal challenges that may occur during this time in children with Cerebral Palsy (CP).

2. What is the purpose of this research?

The purpose of this optional genetic research project is to study how genetic differences may influence the way children with CP develop musculoskeletal problems.

3. What does participation in this research involve?

Your child is being invited to participate in this genetic research sub-study because your child has been diagnosed with CP and is taking part in the main AusCP MSK study.

If you consent to your child participating in optional genetic research, we will collect one additional 4mL blood sample (approximately 1 teaspoon) during the study. The optional genetic study is separate from the mandatory blood testing your child undergoes in the main study.

There are no additional risks involved in this blood collection procedure further to those explained in the main study information and informed consent form.

Your child's additional test samples will be coded like the ones collected for the purposes of the main study and as an added level of security will be labelled with a different code than in the main study before we use them for this research project.

You are being asked for your child to donate a blood sample for genetic research that will look at your child's DNA and compare it with some or all of the personal information collected in this research project.

The study team does not have to conduct this genetic research, or any additional research on your child's sample or DNA.

4. Who is funding the research project?

The study is funded by the Australian Government Medical Research Futures Fund. We may seek additional funding to assist in the completion of the optional genetic sub-study.

5. Compensation

This trial is covered by standard clinical trial insurance. That means you may be entitled to make a claim if you believe your child suffers an injury as a result of their participation in the study. You may request a copy of the terms of this insurance.

There are no additional costs associated with participating in this optional genetic sub-study, nor will you be paid. Any tests and medical care required as part of the research project will be provided to your child free of charge.

6. What if I wish to withdraw from the research project?

My decision whether or not for my child to participate will not prejudice their future relations with *insert site here*. If I decide for my child to participate, I am free to withdraw my consent and to discontinue participation at any time. The decision to withdraw from the study will not affect their routine medical treatment or their relationship with the people treating them.

Your child's participation in this optional genetic sub-study is voluntary. You are entitled to withdraw your consent at any time, without giving a reason and without a negative effect on your child's standard of medical care. If you wish to withdraw, please inform the study team.

Your child may still continue to participate in the main AusCP MSK study even if you choose to withdraw from optional genetic study.

If you withdraw from optional genetic research, your child's coded data and blood sample will not be used for future research and will be destroyed as soon as possible. Your child's coded data (either copied from the main study database or newly generated) will also be destroyed unless this information is already included in analyses or used in scientific publications.

7. What are the possible benefits for my child and other people in the future?

The results of this optional genetic sub-study will not be given to your study team and results will not be put in your medical record as it is for research purposes and is not valid for medical care. You will not receive any direct benefit; however, it may provide valuable information to improve the diagnosis, treatment or care of individuals with CP in the future.

8. Alternative Treatment

There are no alternative treatments, and there will be no negative consequences if you decline to be involved. Your child will get the same care and treatment without being involved in this optional genetic sub-study.

9. What are the possible risks, side effects, discomforts and/or inconveniences?

Please refer to the relevant information in the main AusCP MSK study Parent/Guardian Information and Consent Form for further details. These are no different from those experienced by your child having blood taken in the main study.

Blood tests: taking blood may cause discomfort at the site of the blood test, possible bruising, redness, and swelling around the site, bleeding at the site, feeling of light-headedness when the blood is taken, and rarely, an infection at the site of the blood test or fainting.

10. What will be done to make sure my child's information is confidential?

Your child's coded data and test samples may only be used for scientific health-related research to find new ways to detect, treat, prevent or cure health problems.

They may also be used jointly with information from other sources outside typical clinical research settings, e.g., from public research databases such as the University of Adelaide Genomics Repository. However, they will not be combined with other information in a way that could identify your child.

All blood samples will be securely stored by *insert site here* or by another organisation working with The University of Queensland for up to 15 years and will be destroyed thereafter.

Long-term storage (also called "biobanking") of your child's test samples will be at the *insert site here*, along with the samples of many other people.

"Biobanking" is storing health information and/or blood or tissue for future research studies. Please note that the location of the biosamples may change at the request of the study team. A "biobank" is the place where health information and/or blood or tissue is stored.

Any data generated from your child's blood samples will be stored as long as necessary for scientific research objectives and allowed by law and will be destroyed or made anonymous thereafter.

11. Who can access my child's data and test samples?

The research team will only collect your child's data and test samples after they have been coded (which means that your child's name, contact details, and personal identifying information, have been replaced by a unique study identification number).

The research team may share your child's coded data and test samples with its research partners and others involved in the study testing and analysis for the purposes of this research project.

Your child's coded data and test samples may also be shared with scientific journals, so the study results can be reviewed by independent scientists and to ensure the accuracy of results.

In **none** of these cases will your child's identity be revealed.

Some of the research collaborators and others may be located outside Australia. If this other country does not have equivalent personal data protection standards than Australia, appropriate safeguards (such as contracts and technical security measures) will be adopted to protect and maintain the confidentiality of your child's data and test samples.

12. What will happen when the research project ends?

We may have study coded data and test samples from many people over many years before we can know if the results of optional genetic research are meaningful.

Therefore, you should not expect to receive individual results from this optional genetic research. We will not give any such data to your child's doctor and we will not put them in your child's medical record as they are only for research purposes and not valid for medical care.

There may be an opportunity to be notified by a third party (My Research Results) about information that unexpectedly arises from the research that might be important for your health or the health of your relatives.

13. Who should I contact for more information?

If you would like more information about the project or if you need to speak to a member of the research team in an emergency please contact:

**Name:**	*site PI name*
**Position:**	*site PI position*
**Phone:**	*Site contact phone*
**Email:**	*Site contact email*

All research in Australia involving humans is reviewed by an independent group of people called a Human Research Ethics Committee (HREC). **Reviewing HREC approving this research and HREC Executive Officer details.**

14. HREC Information:

The Children's Health Queensland Hospital and Health Service Human Research Ethics Committee (HREC) has approved this study. If you have any concerns and/or complaints about the project, the way it is being conducted or your child's rights as a research participant and would like to speak to someone independent of the project, please contact the HREC Office.

**Name:**	HREC Coordinator
**Contact telephone:**	(07) 3069 7002
**Email:**	CHQETHICS@health.qld.gov.au

15. Local Governance Contact Information:

**Name:**	Research Governance Officer
**Contact telephone:**	*Local RGO details here*
**Email:**	*Local RGO details here - must be generic address*

Parent/ Guardian Consent Form

**Project Title**	The Australian Cerebral Palsy Musculoskeletal Health Network Study (AusCP MSK)
**Protocol Number**	HREC/2022/QCHQ/87118
**Principal Investigator**	*site PI name*

Declaration by Parent/Guardian

- The study team have explained the study to me comprehensively
- I/We have had the opportunity to discuss the study with the study team and all of my/our questions were answered satisfactorily
- I/We have had an adequate amount of time to consider the study
- I/We understand the purposes, procedures and risks of the research described in the project
- I/We have read and understood all the above information related to the study, or someone has read it to me in a language that I understand
- I/We understand that I will receive a copy of this Parent/Guardian Information and Consent Form once I/we have signed it
- I/We freely agree to my child participating in this research project as described and understand that I am free to withdraw them at any time during the research project without affecting their future health care
- I/We further understand that my child's information collected in this study may be used in future related research.

OPTIONAL

I/We consent to be contacted by a third party (My Research Results) in the event information that unexpectedly arises from the research that might be important for your health or the health of your relatives.	☐ Yes	☐ No

**Name of Child** (please print)	
**Date**	
**Name of Parent/Guardian** (please print)	
**Signature of Parent/Guardian**	
**Date**	

Independent Witness (if requested)

Under certain circumstances (see Note for Guidance on Good Clinical Practice CPMP/ICH/135/95 at 4.8.9) a witness* to informed consent is required.

I have witnessed the receipt of a Patient Information Sheet by the parent/guardian and exchanging of information between the investigator and the parent/guardian about the study.

An auditor witness would optimally discuss the study with the subject and witness the subject signature

**Name of Witness* to Parent/Guardian's Signature** (please print)	
**Signature**	
**Date**	

* Witness is <u>not</u> to be the investigator, a member of the study team or their delegate. In the event that an interpreter is used, the interpreter may <u>not</u> act as a witness to the consent process. Witness must be 18 years or older.

Declaration by Principal Investigator/ Delegated Study Team Member

I have given a verbal explanation of the research project, its procedures and risks and I believe that the parent/guardian has understood that explanation.

**Name of Study Team Member** (please print)	
**Role of Study Team Member**	
**Signature**	
**Date**	
