# Supplementary material for: Study protocol for the Australasian Cerebral Palsy Musculoskeletal Health Network (AusCP MSK) prospective cohort study: early detection of musculoskeletal complications in young children with moderate to severe cerebral palsy (GMFCS III–V)
Source: BMJ Open. 2025 Apr 30;15(4):e095526. doi: 10.1136/bmjopen-2024-095526 (PMC12049940; doi:10.1136/bmjopen-2024-095526)
Supplement: online supplemental file 2 [file bmjopen-15-4-s002.pdf]

Parent / Guardian Information Sheet

Project Title: The Australian Cerebral Palsy Musculoskeletal Health Network

Thank you for taking the time to read this **Parent/Guardian Information Statement and Consent Form**. We would like to ask your child to participate in a research project that is explained below.

It is ok to say no

- The term "we" refers to the Clinicians (Doctors, Physiotherapists) and researchers.
- In addition, your child will also be informed about this project and may be asked to sign a form to agree to participation in this registry.

HREC/ERM Number: 87118

Version Number: 5.0 Date: 19/07/2023

What is an Information Statement?

These pages tell you about the research project. It explains to you clearly and openly all the steps and procedures of the project. The information is to help you decide whether or not you would like your child to take part in the research. Please read this Information Statement carefully.

Before you decide if you want your child to take part or not, you can ask us any questions you have about the project. You may want to talk about the project with your family, friends or healthcare worker/s.

Important things to know

- It is your choice whether or not your child participates in the research. You do not have to agree if you do not want to.
- If you decide you do not want your child to take part, it will not affect the treatment and care your child receives through the *insert HHS* at the *insert site here*.

If you would like your child to take part in the research project, please sign the consent form provided by the Researcher. By signing the consent form, you are telling us that you:

- understand what you have read
- had a chance to ask questions and received satisfactory answers
- consent to your child taking part in the project

We will give you a copy of this information and consent form to keep.

Your withdrawal from the study

You are under no obligation to continue with the research study. You may change your mind at any time about participating in the research. People withdraw from studies for various reasons, and you do not need to provide a reason.

<u>What is the research project about?</u>

This project is for children with cerebral palsy (CP) who have difficulties with movement and posture that could lead to progressive musculoskeletal (muscle and bone) complications. Children with moderate to

severe CP are at risk of hip displacement, developing scoliosis of their spine (a curve) and/or sustaining a bone fracture. Hip displacement occurs when the femoral head (“ball”) moves out of the acetabulum (“hip socket”). Scoliosis is when the spine curves increase. Some children may experience discomfort, muscle tightness, pain and other symptoms when they have severe hip displacement and /or their spine starts to develop scoliosis. Some children with cerebral palsy have fractures of the arms or legs following minimal trauma. This may cause a lot of pain and sometimes the child needs to have surgery to help the fracture heal. Our project aims to find out whether finding musculoskeletal problems earlier can lead to earlier, timely treatments and reduce the chance of them getting worse. Our research team believe that it's better to find a problem early, so we can try to stop it from becoming a much bigger one.

Who is funding the research project?
The study is funded by the Australian Government Medical Research Futures Fund.

What is involved?

The Australian Cerebral Palsy Musculoskeletal Health Network has 4 Aims each with specific information-gathering activities:

AIM 1: To find early markers of onset/progression of musculoskeletal complications including hip, spine and bone health problems.
AIM 2: To work out how changes to the early brain structure (from brain MRIs already conducted), the potential causes of CP and a child’s musculoskeletal health at recruitment has an impact on their disability outcomes (mobility, self-care, pain) at study completion.
AIM 3: To work out the costs of medical and allied health care and your child’s Quality of life (QOL) at 8 to 13 years in comparison to the severity of musculoskeletal problems.
AIM 4: Determine how commonly children with CP break their bones, the costs of treatment and the clinical findings of children with CP who have a broken bone. We aim to develop a way of predicting which children are more likely to have a fracture.

The study team will also ask whether you are willing for your child to participate in an optional genetic sub-study. If you are potentially interested in this sub-study, you will be given a separate Parent/Guardian Information and Consent Form to consider. You can decide for your child to not take part in this sub-study. If you decide you do not want your child to take part in the optional research, your child can still take part in the main study.

Why is my child being asked to take part in the study?

You and your child are asked to participate in this study because:

- your child is between the ages of 4 to 9 years old
- they have a confirmed diagnosis of cerebral palsy, and
- their sitting and walking abilities match Gross Motor Function Classification System (GMFCS) level III, IV or V. This means that they require assistance to stand, sit or walk.

Duration of the research: How long will I take part in this research?

If you and your child agree to participate in this study, you and your child will have testing done during routine clinical visits to the hospital. This study will be run over 4 years, one timepoint per year, with visits at *insert site name here*. We will try to coincide these visits with other appointments your child has at *insert site name here*.

What does my child need to do in this research project?

If enrolled in this study, you and your child will be required to complete four (4) assessments: 1) at the beginning of the study; 2) 1 year after the study began; 3) 2 years after the study began; and 4) 3 years after the study began. This includes 4 visits in total over 4 years.

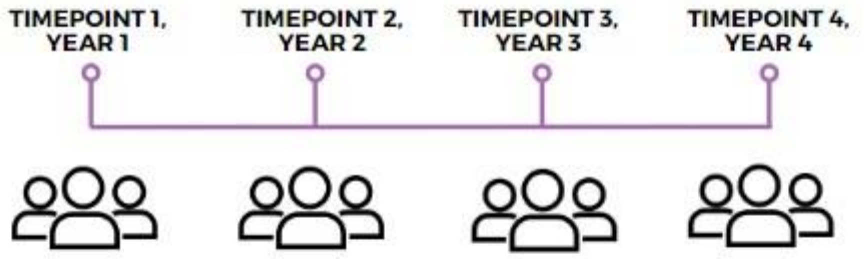

If you agree to participate in the study, there are a number of steps:

We will contact you with a 10–15-minute telephone call to talk about the project and ask you some questions to help us see whether your child is a good fit for the study.

You and your child will be invited to attend the *insert your site here* for your study visits. Whenever possible, information for this study will be collected during your child's regular clinic appointments. All staff undertaking the assessments are registered health practitioners and trained in looking after children with CP.

A) Your child's early medical history

We will ask you about information on your child's early medical and birth history. If your child has been in other clinical trials and you agree, we can retrieve the information from these studies and/or your child's hospital discharge summaries and medical records.

B) Previous brain MRIs and cranial ultrasound scans: We will ask your permission to get copies of your child's previous brain MRI and ultrasounds.

C) Investigations and assessments that your child will undertake at each study visit :

1. Growth: Your child's body weight will be measured using chair scales. We will measure their height using a measuring device called a stadiometer. If they cannot stand by themselves, their length will be measured lying down. Their knee height will also be measured to predict height in children where standing height or supine length may be difficult or impossible to acquire.

2. X-rays of their hip and spine: Your child's hip and spine X-rays will be reviewed and collected with all identifying information removed so that we can take measurements of any hip displacement and scoliosis they may have. The child's hip X-rays will be part of routine screening. Depending on whether your child already has a scoliosis, the spine X-rays may be in addition to what they would normally have done.

3. Physical exam and range of motion evaluation: At each visit, a physiotherapist will take measurements of the amount of movement in your child's hips, knees, feet and spine. These measurements take about 10-15 minutes to do.

4. Bone health:

a. **Bone Mineral Density (BMD) and body composition:** A bone mineral density scan will be done in the 1st, 3rd and potentially the 4th years of the study using Dual-energy X-ray Absorptiometry (DXA, often pronounced 'dexa'). The scan will take images of your child's whole body, lower back, left and right hips, and thighs. For this scan, your child will be laying down and will need to lay reasonably still. To help with this we may ask to loosely wrap your child in a sheet. The bone mineral density and body composition testing will take approximately 45-60 minutes to do.

b. **Peripheral quantitative computer tomography (pQCT):** A second set of images may be taken on a different machine using pQCT. This is another way of assessing bone density

and strength. It is performed on your child's lower leg (tibia, 'shin bone') in the 1st and 3rd years. Your child will place their leg into a small device that helps hold the leg still. If it is not possible to place your child's leg in the device, they will not have to do this scan.

5. Gross Motor Function Measure (GMFM): measures how your child does different physical activities like lying, rolling, sitting, standing, and walking. The test will be recorded by video to allow the physiotherapist to accurately score the GMFM. The GMFM takes about 20 minutes to do and will be completed each year.

6. Classification of function: the following classifications will be assessed at each visit. These classifications are very quick to do and are often done as part of the physical exam or GMFM.

a. **Gross Motor Function Classification System (GMFCS):** The GMFCS records you child's self-initiated movements such as sitting, walking and the use of mobility devices in everyday life.

b. **Manual Ability Classification System (MACS):** The MACS records how your child uses their hands in everyday life.

c. **Communication Function Classification System (CFCS):** The CFCS records how your child communicates.

7. Blood tests: A blood test will be collected by an expert blood collector or under general anaesthetic (if your child is having a procedure under anaesthesia). The blood tests will measure hormones involved in growth, vitamin D, calcium and phosphate levels. These can be altered in children with CP. The blood test will take about 5-10 minutes to do and will be completed each year.

8. Brain structure: If you child has previously had an MRI of their brain taken, the MRI images will be assessed. If they have not had an MRI done before, they will not need to have one done especially for this study.

9. Habitual physical activity: We want to measure your child's levels of physical activity over a 7-day period. To do this we will use small devices with sensors inside that measure movement. We will fit these devices to your child's wrist and thigh at the end of each visit. These sensors are the same as those inside smartwatches. Just like a smartwatch, the devices do not emit anything harmful. They sit on top of the skin and do not cause any pain. Sometimes they can be uncomfortable if too tight, but we will do everything we can to fit them properly so that your child does not experience any discomfort. We clean and sanitise the devices between users. Your child will use the following two devices, where possible:

- The ActiGraph GT3X-wBT: a watch-sized device that is on an elastic watchband. This is worn on their less affected wrist, like a watch.
- Axivity AX3: a smaller device placed on your child's thigh with an adhesive patch.

We will ask your child to wear the device for 7 days. They can be removed during bathing or swimming. You will be asked to complete a log-sheet over the 7 days to record any times you're your child is not wearing the device, and your child's sleep times. We will provide you with a detailed information pack and a return, pre-paid envelope for you to return the devices to us free of charge. This will be completed each year.

D) Questionnaires about your child: We will ask you to complete the following questionnaires. If you would like, some of these questionnaires can be sent out for you to complete 2 weeks prior to any of your child's study visits to minimise the time spent with the study team on the day. Study staff will be available to assist you with any questionnaires or answer any questions you may have.

1. Demographic questionnaire: We will ask questions about your child and family. We will also ask for your permission to access your child's previous medical history and tests. We are interested

in whether your child has had an MRI scan of their brain, or if they have had any previous medical interventions. This questionnaire is completed once, at the first study visit.

2. **Perinatal and birth history questionnaire:** We will ask about your child's genetic history and information regarding your pregnancy and the birth of your child. This questionnaire is completed once, at the first study visit.

3. **Clinical History questionnaire:** We will ask about your child's previous medical history and information, including clinical treatments, age and severity of CP, early intensive therapy interventions, and any investigations they may have had.

4. **Pediatric Evaluation of Disability Inventory (PEDI-CAT):** The questionnaire asks you to rate how your child does a range of daily activities. It asks about their mobility, cognitive and social abilities, and how much assistance you give your child to do everyday activities. You can do this questionnaire on a laptop or tablet. It will take you 15-20 minutes to complete each year.

5. **Fracture questionnaire:** Once a year you will fill in a fracture questionnaire. If your child has a fracture, you will be asked to let us know about it within 24 hours, including information on if/where an X-ray has been completed and what the management details are.

6. **Health resource usage questionnaire:** This questionnaire asks about the types of health care and equipment that your child uses. It will help us to record any costs of health resource use associated with your child's care and musculoskeletal outcomes. This will be completed each year.

7. **Child Health Utility (CHU):** We will ask you to complete a very short questionnaire called the Child Health Utility which asks you to rate 9 short items about your child's health. The CHU will take about 2 minutes to do each year and will give us more of an understanding about the costs and benefits of health care.

8. **Pain experience:** Episodes of pain and any treatments, including the need to see a doctor or use of medications, will be assessed using a survey and scoring tool called the Paediatric Pain Profile. If possible, your child will be asked to rate their pain on a series of smiling faces called the Wong Baker Faces Pain Scale. This will be completed each year.

9. **Pubertal development**: You will be shown a diagram each year called the Tanner Stage of Puberty and asked to circle which diagram best represents your child's pubertal stage.

10. **Nutrition:** We will ask you about your child's diet, feeding and frequency of certain foods over the last 6 months through our parent reported Feeding and Food Frequency Questionnaire. This will help us to work out the vitamin D and calcium your child gets from the food they eat. It includes any gastrostomy feeds for children who have a PEG. This takes about 15 minutes to complete.

    If your child eats some foods orally, you will also be asked to complete the Australia Recommended Feeding Score (AFRS). This takes about 10 minutes to do.

    The nutrition questionnaire is completed each year.

11. **Sun exposure:** For the 7 days after each assessment visit, you will be asked to complete a Sun Diary to record the time your child spends in the sun. This will help us to assess the natural vitamin D they receive. The diary will take about 5-10 minutes to do each day.

12. **The Caregiver Priorities & Child Health Index of Life with Disabilities (CPCHILD©) Questionnaire:** the CPCHILD is a questionnaire that asks you to rate how easy or difficult many activities are across a number of areas in your child's daily life including personal care, positioning, transferring and mobility; their comfort and emotions; and their communication and social interactions. This takes about 15 minutes to do, each year.

We will try to make participating in this study as easy as possible by completing any assessments, scans or tests related to the study at your child's regular hospital appointments.

The total time for assessments with your child is approximately 4 hours at each study visit, and approximately 2.5 hours for you to complete the parent questionnaires. Please note that this time may fluctuate depending on wait-times at departments (i.e., Radiology, Pathology). You will be given the opportunity to complete some of the questionnaires at home prior to assessment visits if you choose. The first timepoint will typically be the longest and most time intensive, though we will make every effort to minimise the time spent on study assessments at each timepoint.

**Schedule of Activities**				
**Study Period**	**Timepoint 1**	**Timepoint 2**	**Timepoint 3**	**Timepoint 4**
**General Procedures**				
Informed Consent	✓			
Inclusion/Exclusion criteria review	✓			
	✓			
**Retrospective Data**				
Perinatal and Birth History	✓			
Clinical History	✓			
**Assessments**				
Anthropometry (height, weight)	✓	✓	✓	✓
X-rays – hip and PA/AP & lateral spine	✓	✓	✓	✓
Musculoskeletal Range of Movement Assessment	✓	✓	✓	✓
Spinal Range of Movement Assessment	✓	✓	✓	✓
DXA	✓		✓	✓
pQCT	✓		✓	✓
Blood test	✓	✓	✓	✓
Genetic Testing (optional) *	✓			
GMFM 88	✓	✓	✓	✓
PEDI-CAT	✓	✓	✓	✓
Maturation	✓	✓	✓	✓
Classifications	✓			
Habitual Physical Activity	✓	✓	✓	✓
Sun Diary	✓	✓	✓	✓
**Parent-Reported Outcome Assessments**				
Demographics Questionnaire	✓			
Health Resource Use Questionnaire	✓	✓	✓	✓
Fracture Questionnaire	✓	✓	✓	✓
Feeding Questionnaire	✓	✓	✓	✓
Paediatric Pain Profile	✓	✓	✓	✓
Child Health Utility 9D (CHU-9)	✓	✓	✓	✓
CP-CHILD	✓	✓	✓	✓

* Completed once at any timepoint in the duration of the study

E) Medicare and Medication Costs

We will capture data on medical and pharmacy services your child uses. You will be asked to sign a consent form from Services Australia allowing us to access your child's Medicare Benefits Schedule (MBS) and/or Pharmaceutical Benefits Scheme (PBS) records from their date of birth to the date of the assessment. Medicare collects information on your child's doctor visits and other medical costs, while the PBS collects information on the prescription medications you have filled at pharmacies.

Services Australia is not involved in this study other than to give us information about your child's Medicare Benefits Schedule (MBS) and/or Pharmaceutical Benefits Scheme (PBS) claims.

Services Australia will not provide your child's personal information to the study without your consent. For this part of the study, we will ask you to complete the 'Services Australia Participant Consent Form'.

The consent form is sent securely to Services Australia who keep MBS and PBS data confidentially.

Because the study involves children under the age of 14 years, a parent must provide consent for their child to participate. If a child is on two separate Medicare cards, there is the option for both primary cardholders to provide consent. If only one parent/carer has provided consent, Services Australia will only provide data that is related to the consenting parent/carer.

What if I wish to withdraw from the research project?
Your decision about whether or not your child participates will not change their future relationship with Health Care Professionals at *insert site here*.

If you decide for your child to participate, you are free to withdraw your consent and to discontinue participation at any time. The decision to withdraw from the study will not affect your child's routine medical treatment or their relationship with the person treating them.

You can withdraw your child from the study at any time by completing and signing the 'Parent/Guardian Withdrawal of Consent Form' provided to you. If you withdraw from the study, you will be able to choose whether the study will destroy or continue to use the information that has been collected about your child. You should only choose one of these options. If you do not tick a box, or tick both boxes accidentally, we will destroy all information that has been collected about your child.

You do not have to continue with the consent to release your child's Services Australia information (Medicare and PBS). You may change your mind at any time. People withdraw from studies for various reasons, and you do not need to provide a reason.

You can withdraw your consent to release your child's Services Australia information by completing and signing the 'Services Australia Participant Withdrawal of Consent Form'. If you withdraw your consent, you will be able to choose whether the study will destroy or keep the Services Australia information it has collected about your child. You should only choose one of these options. If you do not tick a box, or tick both boxes accidentally, we will destroy all information that has been collected about your child. If you do withdraw your consent from the study and your child's information has already been analysed and/or included in a publication, their personal information may not be able to be withdrawn or destroyed. In this case, their information will continue to form part of the study records and results. Your child's privacy will continue to be protected at all times.

Benefits: What are the possible benefits of participation?
You and your child will not benefit directly from participating in this study. What we find out from this study may help to guide future treatment for your child and others. We will share the results of the study with you when it is finished.

The information from this study may help select the best treatments for preventing hip dislocation, scoliosis and low trauma fracture, relieving pain, help caregiving and improving the quality of life of children with moderate to severe CP and their parents/caregivers. The findings will have applications for the care of children with moderate to severe CP across Australia.

Alternatives: What other choices do I have other than participating in the study?
There will be no negative consequences if you decline to be involved. Your child will get the same care and treatment without being involved in the research.

Will I be paid for being in the research?
You will not be paid for taking part in this research. *insert here if applicable: "You will receive free carparking at *site* during your study visits."*

What are the possible risks, side effects, discomforts and/or inconveniences?
There are no potential harms associated with this study. Any risk involved in this study is no greater than your child's usual clinical care. The time taken to do all the tests and questionnaires may be inconvenient for you. The assessments will happen at *insert site name here*, and will be planned to coincide with your child's regular hospital appointments to minimize the need to make a special trip to the hospital. The possible inconvenience to you and your child is the time that the assessments will take, which is approximately 4-6 hours for each visit, 4 visits over 4 years. The assessments might be a bit tiring, so there will be scheduled breaks during the assessment. You will also be able to complete some of the questionnaires in your own time, before or after the assessment visit if you choose.

Your child may experience slight discomfort during the physical exam or X-rays.
The X-rays of the hips will be performed as part of your child's usual clinical care. Depending on your child's clinical status, the spine X-rays and DXA may be additional to X-rays they currently have.

- *Sites to insert site-specific radiation safety assessment information below, OR use the following:*

This research study involves exposure to an amount of radiation. As part of everyday living, everyone is exposed to naturally occurring background radiation and receives a dose of about 2 millisieverts (mSv) each year. The effective dose from this study is about 0.2 mSv. The benefits from the study should be weighed against the possible detrimental effects of the additional radiation exposures, including an increased risk of cancer induction. In this particular study, the risk is minimal, and the estimated risk of such harm is up to about 1 in 10,000. At this dose level, no harmful effects of radiation have been demonstrated as any effect is too small to measure.

What will be done to make sure my child's information is confidential?
- All results and information from the tests, scans, assessments and questionnaires will be stored without your child's name on them.
- A number is used to identify the information. This number is linked to your child's name but the linking file will be kept confidential and only made available to the researchers.
- We will use electronic forms and all information will be kept in a database at the Queensland Cerebral Palsy and Rehabilitation Research Centre, at the University of Queensland. Any paper forms that we use to record the assessments and questionnaires will be stored in a secure filing cabinet and only the researchers will have access to this information. The paper forms containing assessment or questionnaire results will be kept at insert site/institution here* in a locked filing cabinet. *insert if applicable: "Under current Queensland/ *other state* law, no information will be destroyed."*
- Results will be electronically entered into The University of Queensland (UQ) Research Electronic Data Capture (REDCap) platform, which is protected by 2-factor authentication. Access to REDCap will be controlled by UQ.
- Any video recordings of the assessments will be kept at the Queensland Cerebral Palsy and Rehabilitation Centre on a secure University of Queensland server.
- MRI scans will be shared with a member of the research team who works at the Commonwealth Scientific and Industrial Research Organisation (CSIRO) and with other research team members. The CSIRO adheres to strict privacy policies that can be found at https://www.csiro.au/en/about/policies/privacy. Because MRIs are images, they could be potentially re-identifiable. When they are shared, any of your child's personal information on the image will be removed and a code number will be added instead.
- If we give talks or write about the results of this project, we will not use any names or identifying details.
- If you give us permission via signing the participant consent below, we would like to be able to use the visual recordings to educate and train health professionals and researchers on how to conduct the assessments.

- Your child's Medicare Benefits Schedule and Pharmaceutical Benefits Scheme data will remain confidential and will not be disclosed without your permission, except as required by law. You can withdraw your consent later. Your child's coded Medicare Benefits Schedule and Pharmaceutical Benefits Scheme data will be held securely and confidentially by our study team members at the Queensland Cerebral Palsy and Rehabilitation Research Centre to enable us to calculate costs. The Medicare Benefits Schedule and Pharmaceutical Benefits Scheme data cannot be used for any other purposes other than those approved for the study. The cost data from this study will be stored securely for 5 years from the end of the study, after which it will be destroyed securely.

Compensation

This trial is covered by standard clinical trial insurance. That means you may be entitled to make a claim if you believe your child suffers an injury as a result of their participation in the study. You may request a copy of the terms of this insurance.

Research Staff

There may be other experienced health professionals such as radiologists, occupational therapists and physiotherapists who will be trained to undertake assessments at the specific approved sites.

What will happen when the research project ends?

At the completion of the study, families will be provided with a final report of the findings of the study with a summary of results.
Families will also be provided with information on any publications arising from this research project. A list of any publications will be kept updated and able to be found at https://child-health-research.centre.uq.edu.au/research/med-kids.

Who should I contact for more information?

If you would like more information about the project or if you need to speak to a member of the research team in an emergency please contact:

**Name:**	*Sites to enter Site Coordinator information*
**Position:**	*Sites to enter Site Coordinator information*
**Phone:**	*Sites to enter Site Coordinator information*
**Email:**	*Sites to enter Site Coordinator information*

All research in Australia involving humans is reviewed by an independent group of people called a Human Research Ethics Committee (HREC).

HREC Information:

The Children's Health Queensland Hospital and Health Service Human Research Ethics Committee (HREC) has approved this study. If you have any concerns and/or complaints about the project, the way it is being conducted or your child's rights as a research participant and would like to speak to someone independent of the project, please contact the HREC Office.

**Name:**	HREC Coordinator
**Contact telephone:**	(07) 3069 7002
**Email:**	CHQETHICS@health.qld.gov.au

Local Governance Contact Information:

**Name:**	Research Governance Officer
**Contact telephone:**	*Sites to enter local RGO Information*
**Email:**	*Sites to enter local RGO Information*

Services Australia has confirmed that this research and any associated documents have been approved by a Human Research Ethics Committee (HREC) that is registered with the National Health and Medical Research Council (NHMRC) and operates within guidelines set out by the NHMRC.

If you have a privacy complaint in relation to the use of your MBS/PBS data you should contact the Office of the Australian Information Commissioner. You will be able to lodge a complaint with them.

Website: www.oaic.gov.au
Telephone: 1300 363 992
Email: enquiries@oaic.gov.au
Mail: GPO Box 5218, Sydney NSW 2001

Your personal information Services Australia hold is protected by the Privacy Act 1988 and cannot be given to a third party without your consent or where otherwise permitted by law. For more information about privacy, go to **servicesaustralia.gov.au/privacy**

You can withdraw your child from the study at any time by completing and signing the ‘Participant Withdrawal of Consent Form’ that will be provided to you. The decision to withdraw from the study will not affect your child’s routine medical treatment or their relationship with the person treating them.
If you withdraw from the study, you will be able to choose whether the study will destroy or retain the information it has collected about you. You should only choose one of these options. If you do not tick a box, or tick both boxes accidentally, we will destroy all information that has been collected about your child.

Parent/Guardian Consent Form

**Project Title**	AusCP MSK: The Australian Cerebral Palsy Musculoskeletal Health Network
**Protocol Number**	HREC/2022/QCHQ/87118
**Principal Investigator**	*Insert Site PI Name Here*

Declaration by Parent/Guardian:

- The study team have explained the study to me comprehensively
- I/We have had the opportunity to discuss the study with the study team and all of my/our questions were answered satisfactorily
- I/We have had an adequate amount of time to consider the study
- I/We understand the purposes, procedures and risks of the research described in the project
- I/We have read and understood all the above information related to the study, or someone has read it to me in a language that I understand
- I/We understand that I will receive a copy of this Parent/Guardian Information and Consent Form once I/we have signed it
- I/We freely agree to my child participating in this research project as described and understand that I am free to withdraw them at any time during the research project without affecting their future health care
- I/We understand that the name of our family GP will be collected in order to allow direct sharing of information and concerns regarding potential risks for the child if necessary.
- I/We further understand that my child's information collected in this study may be used in future related research.

I/We agree to be contacted in future if a further research study is planned	☐ Yes	☐ No

**Name of Child** (please print)	
**Date**	
**Name of Parent/Guardian** (please print)	
**Signature of Parent/Guardian**	
**Date**	

Under certain circumstances (see Note for Guidance on Good Clinical Practice CPMP/ICH/135/95 at 4.8.9) a witness* to informed consent is required.

Independent Witness (if requested)

I have witnessed the receipt of a Patient Information Sheet by the parent/guardian and exchanging of information between the investigator and the parent/guardian about the study.

An auditor witness would optimally discuss the study with the subject and witness the subject signature

**Name of Witness* to Parent/Guardian's Signature** (please print)	
**Signature**	
**Date**	

* Witness is <u>not</u> to be the investigator, a member of the study team or their delegate. In the event that an interpreter is used, the interpreter may <u>not</u> act as a witness to the consent process. Witness must be 18 years or older.

Declaration by Principal Investigator/ Delegated Study Team Member

I have given a verbal explanation of the research project, its procedures and risks and I believe that the parent/guardian has understood that explanation.

**Name of Study Team Member** (please print)	
**Role of Study Team Member**	
**Signature**	
**Date**	
