# Supplementary material for: Study protocol for the Australasian Cerebral Palsy Musculoskeletal Health Network (AusCP MSK) prospective cohort study: early detection of musculoskeletal complications in young children with moderate to severe cerebral palsy (GMFCS III–V)
Source: BMJ Open. 2025 Apr 30;15(4):e095526. doi: 10.1136/bmjopen-2024-095526 (PMC12049940; doi:10.1136/bmjopen-2024-095526)
Supplement: online supplemental file 3 [file bmjopen-15-4-s003.pdf]

Parent / Guardian Information Sheet

Transiliac Bone Biopsy
(Part of the Project: The Australian CP Musculoskeletal Health Network)

Thank you for taking the time to read this **Parent/Guardian Information** We would like to ask your child to participate in a research project that is explained below.

It is ok to say no

- If you are the parent/guardian of a child under 18 years old who is being asked to be in this study, the word "you" in this document refers to your child. You will be asked to read and sign this document to give permission for your child to participate.
- The term "we" refers to the Clinicians (Doctors, Physiotherapists) and researchers.
- In addition, my child will also be informed about this project and may be asked to sign a form to agree to the participation in this registry.

HREC/ERM Number: 87118

Version Number: 4.0 Date: 31/10/2023

What is an Information Statement?

These pages tell you about the research project. It explains to you clearly and openly all the steps and procedures of the project. The information is to help you decide whether or not you would like your child to take part in the research. Please read this Information Statement carefully.

Before you decide if you want your child to take part or not, you can ask us any questions you have about the project. You may want to talk about the project with your family, friends or health care worker.

If you would like your child to take part in the research project, please sign the consent form provided by the Researcher. By signing the consent form, you are telling us that you:

- understand what you have read
- had a chance to ask questions and received satisfactory answers
- consent to your child taking part in the project

We will give you a copy of this information and consent form to keep.

What is this project about?

This project is for young children with cerebral palsy (CP) who have developed a musculoskeletal (MSK) complication that requires them to undergo an orthopaedic operation. Children with moderate to severe cerebral palsy (CP) are at high risk of dislocating their hips, developing scoliosis of their spine and / or sustaining a low trauma bone fracture. Hip dislocation occurs when the femoral head ("ball") moves out of the acetabulum ("hip socket"). Scoliosis is when the spine curves increase that can lead to muscle tightness, pain and other symptoms. Some children with cerebral palsy sustain fractures of the arms or legs following minimal trauma. Occasionally children with CP need to have orthopaedic surgery on their hip or spine to help reduces pain and stop the hip or spine problem from getting worse.

Who is funding the research project?

The study is funded by the Australian Government Medical Research Futures Fund.

What is involved?

One of the aims of The Australian Cerebral Palsy Musculoskeletal Network is to find out what the bone quality of children with cerebral palsy is like. This is best done by doing a bone biopsy to get a small piece of bone from near the hip at the same time as spine or hip surgery. This piece of bone is then tested to look at the bone's size, structure and density. This information may help us develop better treatments to prevent musculoskeletal complications in children with cerebral palsy.

The study team will also ask whether you are willing for your child to participate in an optional genetic sub-study. If you are potentially interested in this sub-study, you will be given a separate Parent/Guardian Information and Consent Form to consider. You can decide for your child to not take part in this sub-study. If you decide you do not want your child to take part in the optional research, your child can still take part in the main study.

What does my child need to do in this research project?

A) Your child's early medical history

We will ask to get the information on your child's early medical and birth history. If you have been in other clinical trials and you agree, we can retrieve the information from these studies and/or your child's neonatal hospital discharge summaries and electronic medical records.

B) Investigations and assessments your child will undertake:

1. **Transiliac Bone Biopsy**: A transiliac bone biopsy is when a small piece of bone is taken from the ilium, a bone near the hip so that it can be looked at under a microscope.

2. **Growth and nutritional status:** i) body weight using chair scales. ii) Height using a measuring device called a stadiometer, or length using a measuring board your child will lay down on (in children who cannot stand alone). Knee height and upper-arm length will be measured. iii) Body mass index (body weight and height) will be worked out.

    a) **Pubertal development**: Legal guardians/parents will complete the standardised Tanner stage puberty diagrams, to work out the child's pubertal stage.

3. **X-rays of hip or spine:** Your child's routine hip and spine x-rays will be reviewed and collected with all identifying information removed so that we can take measurements of any hip displacement and scoliosis they may have. The child's hip X-rays will be part of routine screening. Depending on whether your child already has a scoliosis, the spine X-rays may be in addition to what they would normally have done.

4. **Physical Exam and Range of Motion Evaluation**. This will involve a physical exam by a physiotherapist to record how much your child can move their arms and legs. Your treating doctor will ask you and/or your child questions about your child's general health.

5. **Bone Health:**

a) **Bone Mineral Density (BMD):** A assessment using Dual-energy X-ray Absorptiometry (DXA) will take simple images of your child's whole body, lower back, left and right hips, and thighs to measure BMD and body composition. For this scan, your child will be laying down and will need to lay reasonably still. To help with this we may ask to loosely wrap your child in a sheet. This bone and body composition testing will take approximately 45 minutes to complete

b) **Peripheral quantitative computer tomography (pQCT):** A second set of images may be taken on a different machine using pQCT. This is another way of assessing bone density and strength. It is performed on the tibia (shin bone). Your child will place their leg into a small device that helps hold the leg still. It may not be possible to place your child's leg in the device. If this is the case, your child will not have to have this test undertaken. This test takes about 20 minutes to complete.

6. **Gross Motor Function Measure (GMFM):** measures how your child does different physical activities like lying, rolling, sitting, standing, and walking. The test takes about 20 minutes to do.

7. **Classification of function:** the following classifications will be assessed at each visit. These classifications are very quick to do and are often done as part of the physical exam or GMFM

   a) **Gross Motor Function Classification System (GMFCS):** The GMFCS records you child's self-initiated movements such as sitting, walking and the use of mobility devices in everyday life.

   b) **Manual Ability Classification Scale (MACS):** works out how well your child uses their hands in everyday life.

   c) **Communication Function Classification System (CFCS):** The CFCS records how your child communicates.

8. **Blood tests:** A blood test will be collected by an expert blood collector or under general anaesthetic (if your child is having a procedure under anaesthesia). The blood tests will measure hormones involved in growth, vitamin D, calcium and phosphate levels. These can be altered in children with CP. The blood test will take about 5-10 minutes to do.

C) Questionnaires About Your Child: The following questionnaires will be completed by the parent or Guardian:

1. **Fracture questionnaire:** We will ask you about your child's bone fracture history in the last 12 months.

2. **Nutrition:** We will ask you about your child's diet, feeding and frequency of certain foods over the last 6 months through our parent reported Feeding and Food Frequency Questionnaire and the Australia Recommended Feeding Score (AFRS). This takes about 15 minutes to do.

3. **Pain experience:** Episodes of pain and any treatments, including the need to see a doctor or use of medications, will be assessed using a survey and scoring tool called the Paediatric Pain Profile. If possible, your child will be asked to rate their pain on a series of smiling faces called the Wong Baker Faces Pain Scale.

Schedule of Activities		
**Study Period**	**Timepoint 1**	**Surgery date**
**General Procedures**		
Informed Consent [a, b]	X	
Inclusion/Exclusion criteria review [a]	X	
**Retrospective Data**		
Clinical History [c, d]	X	
Perinatal and Birth History [c, d]	X	
**Assessments**		
Anthropometry	X	
X-rays – hip and PA/AP & lateral spine	X	
Lower Limb Assessment	X	
Spinal ROM Assessment	X	
Densitometry – DXA[e]	X	
Densitometry – pQCT [f]	X	
Pathology	X	
Genetic Testing (optional) [g]	X	
GMFM-88	X	
Maturation [i]	X	
Classifications [j]	X	
**Parent-Reported Outcome Assessments**		
Fracture Questionnaire [c]	X	
Feeding Questionnaire [c]	X	
Paediatric Pain Profile	X	
**Clinical Procedures**		
Bone biopsy		X

What does having a Transiliac Bone Biopsy involve?

The biopsy will be done at Queensland Children's Hospital under a general anaesthetic at the same time that your child is having surgery on their hip or spine. We will only collect the biopsy on children who have the area that we want to take the piece of bone from already exposed as part of their planned surgical procedure, that is, we will not be doing any extra skin incisions to perform the biopsy. The 5mm piece of bone that we will use for the bone biopsy will have been taken as part of the procedure.

Before the biopsy:

On days 17, 16, 5 and 4 before the biopsy, it is very important that the antibiotic Demeclocycline is taken twice a day as we have put on the Labelling Sheet. This medication is taken to give information on the way bone is made. If the medication is not taken as recommended, the biopsy is less useful.

Day of the biopsy:

On the day of the bone biopsy, your child will get ready for surgery as advised by their treating orthopaedic surgeon. With your permission, and that of your child's surgeon, Prof Craig Munns will attend the operating theatre and take a small piece of bone (5 mm or the size of the top of a pen) from the top of the hip (ilium bone). This does not affect the strength of the ilium bone. It will not interfere with the orthopaedic surgery your child is having done.

After the biopsy:

Your child will go to the ward under the care of the orthopaedic team.

When will we know the result of the Transiliac Bone Biopsy?

The piece of bone will be sent to Prof Peter Pivonka at the Queensland University of Technology. Prof Pivonka and his team will perform a number of tests on the bone. Because these tests are very special, it may take up to 12 months to get the full results back. We will discuss the results of the biopsy with you once they are available.

Your child's early medical history

We will ask to get the information on your child's early medical and birth history. If you have been in other clinical trials and you agree, we can retrieve the information from these studies and/or your child's neonatal hospital discharge summaries and electronic medical records.

Benefits: What are the possible benefits of participation?

You and your child will not benefit directly from being involved in this study. What we find from this study may help guide future treatment decisions for your child and others. We will share the results of the study with you when it is done.

The results we find may help select the best treatments for preventing hip dislocation, scoliosis, and low trauma fracture; relieving pain, helping caregiving and improving the quality of life of children with cerebral palsy and their parents/caregivers.

Alternatives: What other choices do I have other than participating in the bone biopsy study?

Another option is to get the same treatment without being involved in the research. There will be no negative consequences if you and your child decide not to be involved.

What are the possible complications of a Transiliac Bone Biopsy?

The possible inconvenience to you and your child is the time that the assessments will take, which is approximately 4-6 hours. The assessments might be a bit tiring, so there will be scheduled breaks during the assessment. You will also be able to complete some of the questionnaires in your own time, before or after the assessment visit if you choose.

A transiliac bone biopsy will not add any risk to the surgery your child is already having. When other children are admitted for a bone biopsy alone, it is done as day surgery, with the child going to school the next day.

Your child may experience slight discomfort during the physical exam or X-rays. The X-rays of the hips will be performed as part of your child's usual clinical care. Depending on your child's clinical status, the spine X-rays and DXA may be additional to X-rays they currently have.

This research study involves exposure to an amount of radiation. As part of everyday living, everyone is exposed to naturally occurring background radiation and receives a dose of about 2 millisieverts (mSv) each year. The effective dose from this study is about 0.058 mSv. The benefits from the study should be weighed against the possible detrimental effects of the additional radiation exposures, including an increased risk of cancer induction. In this particular study, the risk is minimal, and the estimated risk of such harm is up to about 1 in 10,000. At this dose level, no harmful effects of radiation have been demonstrated as any effect is too small to measure.

Confidentiality

All results of the bone biopsy will be stored without your child's name on them. A number is used to identify the assessments. When the biopsy is sent to Prof Pivonka, he will not know the name of your

child, only the study number. This number is linked to your child's name, but the linking file will be kept confidential and only made available to the researchers. This will allow us to give you back the result of your child's bone biopsy.

- All results and information from the tests, scans, assessments and questionnaires will be stored without your child's name on them.
- A number is used to identify the information. This number is linked to your child's name but the linking file will be kept confidential and only made available to the researchers.
- We will use electronic forms and all information will be kept in a database at the Queensland Cerebral Palsy and Rehabilitation Research Centre, at the University of Queensland. Any paper forms that we use to record the assessments and questionnaires will be stored in a secure filing cabinet and only the researchers will have access to this information. The paper forms containing assessment or questionnaire results will be kept at the Centre for Children's Health Research in a locked filing cabinet.
- Results will be electronically entered into The University of Queensland (UQ) Research Electronic Data Capture (REDCap) platform, which is protected by 2-factor authentication. Access to REDCap will be controlled by UQ.
- If we give talks or write about the results of this project, we will not use any names or identifying details.

Compensation

This trial is covered by standard clinical trial insurance. That means you may be entitled to make a claim if you believe your child suffers an injury as a result of their participation in the study. You may request a copy of the terms of this insurance.

What if I wish to withdraw from the research project?

Your decision on whether or not your child participates will not prejudice their future relations with Health Care Professionals at Queensland Health.

If you decide for your child to participate, you are free to withdraw your consent and discontinue participation at any time. The decision to withdraw from the study will not affect your child's routine medical treatment or their relationship with the person treating them.

You can withdraw your child from the study at any time by completing and signing the 'Participant Withdrawal of Consent Form'. This form is provided at the end of this document and is to be completed by you and supplied to the research team if you choose to withdraw at a later date. If you withdraw from the study, you will be able to choose whether the study will destroy or retain the information it has collected about you. You should only choose one of these options. Where both boxes are ticked in error or neither box is ticked, the study will destroy all information it has collected about you.

Research Staff

There may be other experienced health professionals such as radiologists, occupational therapists and physiotherapists who will be trained to undertake assessments at the specific approved sites.

What will happen when the research project ends?

At the completion of the study, families will be provided with a final report of the findings of the study with a summary of results.

Families will also be provided with information on any publications arising from this research project. A list of any publications will be kept updated and able to be found at https://child-health-research.centre.uq.edu.au/research/med-kids.

Who should I contact for more information?

If you would like more information about the project or if you need to speak to a member of the research team in an emergency please contact:

**Name:**	*Sites to enter Site Coordinator information*
**Position:**	*Sites to enter Site Coordinator information*
**Phone:**	*Sites to enter Site Coordinator information*
**Email:**	*Sites to enter Site Coordinator information*

All research in Australia involving humans is reviewed by an independent group of people called a Human Research Ethics Committee (HREC).

HREC Information:

The Children's Health Queensland Hospital and Health Service Human Research Ethics Committee (HREC) has approved this study. If you have any concerns and/or complaints about the project, the way it is being conducted or your child's rights as a research participant and would like to speak to someone independent of the project, please contact the HREC Office.

**Name:**	HREC Coordinator
**Contact telephone:**	(07) 3069 7002
**Email:**	CHQETHICS@health.qld.gov.au

Local Governance Contact Information:

**Name:**	Research Governance Officer
**Contact telephone:**	*Sites to enter local RGO Information*
**Email:**	*Sites to enter local RGO Information*

Parent/Guardian Consent Form

**Project Title**	AusCP MSK: The Australian Cerebral Palsy Musculoskeletal Health Network
**Protocol Number**	HREC/2022/QCHQ/87118
**Principal Investigator**	*Insert Site PI Name Here*

Declaration by Parent/Guardian:

- The study team have explained the study to me comprehensively
- I/We have had the opportunity to discuss the study with the study team and all of my/our questions were answered satisfactorily
- I/We have had an adequate amount of time to consider the study
- I/We understand the purposes, procedures and risks of the research described in the project
- I/We have read and understood all the above information related to the study, or someone has read it to me in a language that I understand
- I/We understand that I will receive a copy of this Parent/Guardian Information and Consent Form once I/we have signed it
- I/We freely agree to my child participating in this research project as described and understand that I am free to withdraw them at any time during the research project without affecting their future health care
- I/We understand that the name of our family GP will be collected in order to allow direct sharing of information and concerns regarding potential risks for the child if necessary.
- I/We further understand that my child's information collected in this study may be used in future related research.

I/We agree to be contacted in future if a further research study is planned	☐ Yes	☐ No

**Name of Child** (please print)	
**Date**	
**Name of Parent/Guardian** (please print)	
**Signature of Parent/Guardian**	
**Date**	

Under certain circumstances (see Note for Guidance on Good Clinical Practice CPMP/ICH/135/95 at 4.8.9) a witness* to informed consent is required.

Independent Witness (if requested)

I have witnessed the receipt of a Patient Information Sheet by the parent/guardian and exchanging of information between the investigator and the parent/guardian about the study.

An auditor witness would optimally discuss the study with the subject and witness the subject signature

**Name of Witness* to Parent/Guardian's Signature** (please print)	
**Signature**	
**Date**	

* Witness is not to be the investigator, a member of the study team or their delegate. In the event that an interpreter is used, the interpreter may not act as a witness to the consent process. Witness must be 18 years or older.

Declaration by Principal Investigator/ Delegated Study Team Member

I have given a verbal explanation of the research project, its procedures and risks and I believe that the parent/guardian has understood that explanation.

**Name of Study Team Member** (please print)	
**Role of Study Team Member**	
**Signature**	
**Date**	
