# Supplementary material for: Study protocol for the Australasian Cerebral Palsy Musculoskeletal Health Network (AusCP MSK) prospective cohort study: early detection of musculoskeletal complications in young children with moderate to severe cerebral palsy (GMFCS III–V)
Source: BMJ Open. 2025 Apr 30;15(4):e095526. doi: 10.1136/bmjopen-2024-095526 (PMC12049940; doi:10.1136/bmjopen-2024-095526)
Supplement: Supplementary file 4 [file bmjopen-2024-095526.reviewer_comments.pdf]

PEER REVIEW HISTORY

BMJ Open publishes all reviews undertaken for accepted manuscripts. Reviewers are asked to complete a checklist review form and are provided with free text boxes to elaborate on their assessment. These free text comments are reproduced below.

ARTICLE DETAILS

Title (Provisional)

Study Protocol for The Australasian Cerebral Palsy Musculoskeletal Health Network (AusCP MSK) Prospective Cohort Study: Early Detection of Musculoskeletal Complications in Young Children with Moderate to Severe Cerebral Palsy (GMFCS III-V).

Authors

Munns, Craig; Bentley, Laura; Boyd, Roslyn N; Brookes, Denise; Taylor, Maddison J; Pivonka, Peter; Nassar, Natasha; Trost, Stewart; Little, Judith Paige; Tucker, Kylie; Burns, Joshua; Sakzewski, Leanne; Badawi, Nadia; Ware, Robert S.; Comans, Tracy; Willoughby, Kate L; Paget, Simon; Oftedal, Stina; Keramat, Afroz; Bell, Kristie; Copeland, Lisa; Edwards, Priya; Wynter, Meredith; McGowan, Timothy; Goodwin, Bruce; Reddan, Tristan; Briody, Julie; Pagnozzi, Alex; Ebeling, Peter; Milat, Frances; Simm, Peter; Stewart, Kirsty; Center, Jacqueline R; Smithers-Sheedy, Hayley; McKenzie, Anne; Stott, N Susan; Sorhage, Alexandra; Hofman, Paul; Wilson, Nichola; Hermith-Ramirez, Diana; Rutz, Erich

---

VERSION 1 - REVIEW

**Reviewer**	**1**
**Name**	**Chagas, Paula**
**Affiliation**	**Universidade Federal de Juiz de Fora**
**Date**	**28-Nov-2024**
**COI**	**None**

This manuscript details a comprehensive follow-up study on the determinants of musculoskeletal complications related to CP in these children's first years of life. The results will entail a deep understanding of the factors that contribute to these occurrences and highlight important actions for prevention and treatment options.

I am favorable to accepting this paper for publication.

I have minor considerations, most related to typo:

Line 35-38 - review the beginning and end of brackets;

Line 11 - typo: foetal

Line 75 - 10 degrees (the symbol is wrong)

LIne 91 - 90 degrees (the symbol is wrong)

Line 114 - 40 degrees (the symbol is wrong)

Line 201: define GMFM first

Line 293-294 - add to the text the ROM that will be measured and how do you plan for them to be reliable

Line 311 - typo 90 degrees

On the primary and secondary outcomes, some of the measures, the authors describe how they will be applied, others not - please add to all of them how they will be used in the study and how many times.

LIne 345-346: are you referring to Mini-MACS? If so - add the reference.

Lines 355-358: EDACS - it wasn't refereed as an outcome before - please recheck or withdraw.

Line 444: define DRGs, IP and OP

In Table 1 = please check the age of the children = 1-5? or 1-4? and match it to Figure 1.

At the same table, are you using PEDI or PEDI-CAT? Please recheck.

And at Figure 1: Please change GMFCS 1 to GMFCS I

**Reviewer**	**2**
**Name**	**Chen, Brian Po-Jung**
**Affiliation**	**Chang Gung Memorial Hospital Linkou**
**Date**	**12-Dec-2024**
**COI**	**None**

Thank you for the opportunity to review this study protocol. I appreciate the authors' dedication to their thorough systematic review of the existing literature on hip displacement, scoliosis, and skeletal fragility, particularly with the inclusion of extensive population studies. The reviews in the introduction section nicely reinforce and clarify the rationale for conducting the proposed study for this surveillance. Overall, the manuscript clearly articulates the protocol.

That said, I have a few comments regarding areas that may benefit from clarification and potential formatting adjustments. I am happy to continue the discussion with the authors during the revision process to help refine this manuscript and ensure its success.

Comment 01

Line 13-18 (ABSTRACT; Page 5)

"... population-based surveillance of children with moderate to severe functional mobility limitations (Gross Motor Function Classification System [GMFCS] levels III- V)..."

[1]

According to a previously published cohort study (n=374) based on the Victorian Cerebral Palsy Register (Soo et al., 2006), the incidence of hip displacement (i.e., MP > 30%) was 15.1%, 41.3%, 69.2%, and 89.7% for individuals with GMFCS levels II, III, IV, and V, respectively. Although the incidence for individuals with GMFCS level II was low (15.1%), it was not negligible. This proposed study aims to *"explore the early biomarkers of, and interactions between, musculoskeletal complications related to CP, including hip displacement..."* As the authors stated, *"Musculoskeletal complications related to CP for non-ambulant children, including hip displacement, scoliosis and skeletal fragility are often severe and progressive, with a life-long impact."* Individuals with GMFCS level II are often overlooked regarding potential hip displacement issues due to their relatively lower risk and higher gross motor function. Were there specific reasons the authors decided to exclude individuals in level II?

[2]

As the manuscript title indicates, this AusCP MSK study focuses on *“young children with moderate to severe cerebral palsy.”* However, in **Lines 42** and **Line 236**, the inclusion criteria for Cohort B specify CP GMFCS levels I–V, encompassing all functional abilities rather than only “moderate to severe” individuals. While I understand that the purposes differ between Cohort B (levels I–V) and Cohort C (levels III–V), this could be confusing for readers. It might be clearer if the authors omit references to GMFCS levels in the background/introduction sections and instead address them later in the methods section, specifically when describing the different cohorts, as done in the Study Design section (**Lines 222–247**).

Comment 02

Line 164 & Line 511-512

“Aus-CP-CTN” / “Aus-CP-CTN CRE”

The abbreviation is written as “AusCP” without a hyphen in most instances. The authors might consider ensuring consistency throughout the manuscript unless “Aus-CP” and “AusCP” are intended to serve different purposes.

Comment 03

Line 184-186

" predict the severity and rate of progression of musculoskeletal complications... at 8-13 years"

"... on disability outcomes at 8-13 years of age."

"... poor quality of life at 8-13 years of age... at 4-9 years of age."

, etc.

Line 190-195

"... and musculoskeletal development at 1-4 years of age on disability outcomes at 8-13 years of age."

"... poor quality of life at 8-13 years of age, related to severity of musculoskeletal complications at 4-9 years of age."

Could the authors elaborate on how these age ranges (e.g., 1–4 years, 4–9 years, 8–13 years) were determined? Unless I missed some crucial information, it would be helpful if the authors could explain the rationale behind these decisions and their potential relationship with the two time frames the study aims to address.

Comment 04

Line 200-221, 385, 415, 466, and 490

"... lead to poorer MSK outcomes..."

"... predict severity of MSK complications..."

"... predict severity of MSK outcomes..."

"... greater adverse MSK biomarkers..."

"... progression of MSK complications..."

, etc.

The abbreviation "MSK" apparently refers to "musculoskeletal," as in "AusCP MSK," but it is used multiple times only in the study aims section and not elsewhere in the manuscript. The authors might consider writing this term in full as "musculoskeletal" instead of using "MSK," as it is not a commonly used abbreviation.

Comment 05

Line 201

"... such as GMFM,..."

The abbreviation "GMFM" is used here for the first time. Its full form should be provided.

Comment 06

Line 205

"... motor capacity (GMFM-88) at 4-9 years of age..."

Line 326

"The GMFM-88 version is..."

Why did the authors choose to collect GMFM-88 instead of GMFM-66? For the primary target study participants (moderate to severe CP; GMFCS levels III–V), the dimensional scores for the first 2 or 3 dimensions of GMFM are highly likely to be very low or even 0. Do the authors intend to perform all 88 items in GMFM-88, or will they use the basal-ceiling technique to exclude

the items with a score of 0?

Comment 07

Line 231-234

"Cohort A (n=500)..."

According to the *Eligibility Criteria* in **Lines 250–252**, Cohort A must target children with CP in GMFCS levels III–V. For the sake of consistency in statement format, it would be helpful to state this explicitly, as it is later done for Cohort B (GMFCS I – V) in **Line 236** and Cohort C (GMFCS III- V) in **Line 242**.

Comment 08

Line 252-253 & Line 266-267

"(ii) aged between 4.00 to 9.99 years of age at the time of recruitment (<18.00 years of age for Cohort C);"

I found this criterion slightly difficult to read. Perhaps it could be rewritten as *"(ii) aged from 4.00 to 9.99 years (for Cohort A) or from 4.00 to 17.99 years (for Cohort C) at the time of recruitment;"* or something similar. Additionally, I am unsure whether the lower age limit for Cohorts B and C should be 4.00 or another value.

Comment 09

Line 273

"... will be retrieved from the NSW/ACT CP Register..."

The full forms of "NSW" and "ACT" should be provided here but not in **Lines 258–259**.

Comment 10

Line 278

"... MACS, (CFCS) and..."

Brackets around CFCS are not necessary here.

Comment 11

Line 289

"... will involve annual AP pelvis..."

The full form of "AP" should be provided here but is not necessary in **Lines 296–297**.

Comment 12

Line 290-291

"Reimers migration percentage, acetabular index..."

Include the abbreviations "MP" and "AI" here, as the abbreviations for other measurements are stated in the same sentence.

Comment 13

Line 293 & Line 302

"... all AP pelvis X- rays (J Little)."

"... for rotational deformity (J Little)."

I assume "(J Little)" in these two lines refers to one of the co-authors, but what is its purpose?

Comment 14

Line 293-294

"... will include 12 lower limb assessments..."

Which 12 assessments are the authors referring to?

Comment 15

Line 299

"... X-rays including frontal Cobb angle (Cobb angle),..."

I believe it is unnecessary to provide "Cobb angle" as the abbreviation for Cobb angle.

Comment 16

Line 317 & 323

"... will be acquired at T1 and T3,

and at T4..." "... at 3 timepoints

(T1, T3, T4)..."

Why is DXA not required at T2?

Comment 17

Line 379

"... by study investigators at CSIRO using..."

The full form of CSIRO, "Commonwealth Scientific and Industrial Research Organisation," may need to be provided here.

VERSION 1 - AUTHOR RESPONSE

Reviewer 1

Reviewer Comments	Response to Review
This manuscript details a comprehensive follow-up study on the determinants of musculoskeletal complications related to CP in these children's first years of life. The results will entail a deep understanding of the factors that contribute to these occurrences and highlight important actions for prevention and treatment options.<br>I am favorable to accepting this paper for publication.<br>I have minor considerations, most related to typo:	We thank the reviewer for their feedback and have addressed their comments below. To ensure clarity, colour coding has been incorporated. Purple text refers to existing text in the manuscript and green text refers to new additions based on the feedback from the reviewer.
Line 35-38 - review the beginning and end of brackets;	Thank you, brackets have been removed.
Line 11 - typo: foetal	Foetal is Australian English spelling. Have amended spelling to 'fetal'.
Line 75 - 10 degrees (the symbol is wrong)	Thank you, have now amended.
Line 91 - 90 degrees (the symbol is wrong)	Thank you, have now amended.
Line 114 - 40 degrees (the symbol is wrong)	Thank you, have now amended.
Line 201: define GMFM first	Thank you, have now amended.
Line 293-294 - add to the text the ROM that will be measured and how do you plan for them to be reliable	We thank the reviewer for their comment. The manuscript currently states that "The physical examination will measure ROM of the hip, knee and ankle" however we have now extended the sentence to provide

	evidence of reliability estimates: " The physical examination will measure ROM of the hip (flexion, extension, abduction, rotation), knee (extension, flexion) and ankle (dorsiflexion) and this measure has demonstrated good interrater [1, 2], intra-rater[1] and test-rest reliability[3] in children with CP."
Line 311 - typo 90 degrees	Thank you, have now amended.
On the primary and secondary outcomes, some of the measures, the authors describe how they will be applied, others not - please add to all of them how they will be used in the study and how many times.	We have aimed to describe each measure in sufficient detail and to outline how it will be applied to the study. If the reviewer could provide more specific information on which measures they are referring to, we would be happy to address this further.
Line 345-346: are you referring to Mini-MACS? If so - add the reference.	For the prospective data collection (Cohort A), children are aged from 4 – 9 therefore, the MACS will be used. We acknowledge that for retrospective data, the Mini-MACS may have been collected and have added in a sentence and reference to support this: **Manual Ability Classification System (MACS)** is a 5-level system developed for children aged 4-18 years with good interrater reliability ~~in children as young as 2 years on the Early MACS~~ ~~[4]~~[105, 106].
Lines 355-358: EDACS - it wasn't refereed as an outcome before - please recheck or withdraw.	We would like to clarify that the section referencing EDACS (Lines 355–358) is listed under the subheading 'Classifications,' which is nested under the primary outcomes heading. This placement indicates that EDACS was described as part of the classification systems used in the study rather than as a standalone primary outcome.
Line 444: define DRGs, IP and OP	Thank you, have now amended.
In Table 1 = please check the age of the children = 1-5? or 1-4? and match it to Figure 1.	Table 1 describes the retrospective data collection as 1-5 years and the prospective data collection from 4-9 years. In Figure 1, within the Cohort A branch, the retrospective box has been updated to reflect the different ages: "3 years retrospective clinical data (routine care/ previous research), aged 1-5 years".
At the same table, are you using PEDI or PEDI-CAT? Please recheck.	We thank the reviewer for identifying this and have corrected the table to "PEDI-CAT".
And at Figure 1: Please change GMFCS 1 to GMFCS I	Thank you, have now amended.

Reviewer 2

Reviewer Comments	Response to Review
Thank you for the opportunity to review this study protocol. I appreciate the authors' dedication to their thorough systematic review of the existing literature on hip displacement, scoliosis, and skeletal fragility, particularly with the inclusion of extensive population studies. The<br>reviews in the introduction section nicely reinforce and clarify the rationale for conducting the proposed study for this surveillance. Overall, the manuscript clearly articulates the protocol. That said, I have a few comments regarding areas that may benefit from clarification and potential formatting adjustments. I am happy to continue the discussion with the authors during the revision process to help refine this manuscript and ensure its success.	We thank the reviewer for their feedback and have addressed their comments below. To ensure clarity, colour coding has been incorporated. Purple text refers to existing text in the manuscript and green text refers to new additions based on the feedback from the reviewer.
**Comment 01**<br>**Line 13-18 (ABSTRACT; Page 5)**<br>"... population-based surveillance of children with moderate to severe functional mobility limitations (Gross Motor Function Classification System [GMFCS] levels III- V)..."<br><br>[1] According to a previously published cohort study (n=374) based on the Victorian Cerebral Palsy Register (Soo et al., 2006), the incidence of hip displacement (i.e., MP > 30%) was 15.1%, 41.3%, 69.2%, and 89.7% for individuals with GMFCS levels II, III, IV, and V, respectively. Although the incidence for individuals with GMFCS level II was low (15.1%), it was not negligible. This proposed study aims to "explore the early biomarkers of, and interactions between, musculoskeletal complications related to CP, including hip displacement..." As the authors stated, "Musculoskeletal complications related to CP for non-ambulant children, including hip<br>displacement, scoliosis and skeletal fragility are often severe and progressive, with a life-long impact." Individuals with GMFCS level II are often overlooked regarding potential hip displacement issues due to their relatively lower risk and higher gross motor function.	We thank the reviewer for their comment. The authors acknowledge that it is important to explore the musculoskeletal problems in children with GMFCS level II, however the focus of this study is on the musculoskeletal problems of children with moderate-severe CP, commonly grouped as GMFCS III-V in the literature.

Were there specific reasons the authors decided to exclude individuals in level II?	
As the manuscript title indicates, this AusCP MSK study focuses on "young children with moderate to severe cerebral palsy." However, in **Lines 42** and **Line 236**, the inclusion criteria for Cohort B specify CP GMFCS levels I–V, encompassing all functional abilities rather than only<br>"moderate to severe" individuals. While I understand that the purposes differ between Cohort B (levels I–V) and Cohort C (levels III–V), this could be confusing for readers. It might be clearer if the authors omit references to GMFCS levels in the background/introduction sections and instead address them later in the methods section, specifically when describing the different cohorts, as done in the Study Design section (**Lines 222–247**).	Thank you for this feedback. We appreciate your observation regarding the potential for confusion around the inclusion criteria and the use of GMFCS levels in our manuscript. However, we believe it is important to retain references to GMFCS levels in the introduction/background sections, as these sections discuss prior research findings where GMFCS levels are a critical contextual factor. Removing such references could detract from the relevance and clarity of the literature review and the rationale for the study.
**Comment 02**<br>**Line 164 & Line 511-512**<br>"Aus-CP-CTN" / "Aus-CP-CTN CRE"<br>The abbreviation is written as "AusCP" without a hyphen in most instances. The authors might consider ensuring consistency throughout the manuscript unless "Aus-CP" and "AusCP" are intended to serve different purposes.	The Aus-CP-CTN and Aus-CP-CTN CRE are separate entities from the AusCP MSK study and are incorporated under that hyphenated title with the intention to serve different purposes.
**Comment 03**<br>**Line 184-186**<br>" predict the severity and rate of progression of musculoskeletal complications... at 8-13 years"<br>"... on disability outcomes at 8-13 years of age."<br>"... poor quality of life at 8-13 years of age... at 4-9 years of age."<br>, etc.<br><br>**Line 190-195**<br>"... and musculoskeletal development at 1-4 years of age on disability outcomes at 8-13 years of<br>age."<br>"... poor quality of life at 8-13 years of age, related to severity of musculoskeletal complications at 4-9 years of age."<br><br>Could the authors elaborate on how these age ranges (e.g., 1–4 years, 4–9 years, 8–13 years) were determined? Unless I missed	Musculoskeletal problems often begin in late childhood into early adolescence. The aim of this study is to pick up the earliest biomarkers of musculoskeletal change, hence the decision to recruit children earlier at 4-9 and tracking them over 4 years, when they are 8-13. While we are prospectively assessing musculoskeletal development from age 4, we acknowledge that there might be some important features of musculoskeletal health which contribute to our understanding of early biomarkers before the age of 4 which we can collect retrospectively.

some crucial information, it would be helpful if the authors could explain the rationale behind these decisions and their potential relationship with the two time frames the study aims to address.	
**Comment 04**<br>**Line 200-221, 385, 415, 466, and 490**<br>"... lead to poorer MSK outcomes..."<br>"... predict severity of MSK complications..."<br>"... predict severity of MSK outcomes..."<br>"... greater adverse MSK biomarkers..."<br>"... progression of MSK complications..."<br>, etc.<br><br>The abbreviation "MSK" apparently refers to "musculoskeletal," as in "AusCP MSK," but it is used multiple times only in the study aims section and not elsewhere in the manuscript.<br><br>The authors might consider writing this term in full as "musculoskeletal" instead of using "MSK," as it is not a commonly used abbreviation.	Have amended all singular uses of MSK to musculoskeletal in manuscript and tables.
**Comment 05**<br>**Line 201**<br>"... such as GMFM,..."<br>The abbreviation "GMFM" is used here for the first time. Its full form should be provided.	Thank you, have now amended.
Comment 06<br>Line 205<br>"... motor capacity (GMFM-88) at 4-9 years of age..."<br>Line 326<br>"The GMFM-88 version is..."<br><br>Why did the authors choose to collect GMFM-88 instead of GMFM-66? For the primary target study participants (moderate to severe CP; GMFCS levels III–V), the dimensional scores for the first 2 or 3 dimensions of GMFM are highly likely to be very low or even 0. Do the authors intend to perform all 88 items in GMFM-88, or will they use the basal-ceiling technique to exclude the items with a score of 0?	For the Cohort A participants (moderate to severe CP; GMFCS levels III–V), the dimensional scores for the first 2 or 3 dimensions of GMFM are highly likely to be very low or even 0. We intend to perform all 88 items in GMFM-88 and then from that data analyse the GMFM-66. Performing all items of the GMFM-88 will enable analysis by domain (i.e. Lying, sitting etc) in children who are GMFCS III-V.
**Comment 07**<br>**Line 231-234**<br>"Cohort A (n=500)..."<br>According to the Eligibility Criteria in **Lines 250–252,** Cohort A must target children with CP in GMFCS levels III–V. For the sake of	We thank the reviewer for this feedback and have amended to read: "Cohort A (n=500) involves the collection of three years retrospective data and subsequent 4-year prospective follow-up (baseline (T1) and 3 annual assessments (T2, T3, T4)) for a range

consistency in statement format, it would be helpful to state this explicitly, as it is later done for Cohort B (GMFCS I – V) in **Line 236** and Cohort C (GMFCS IIIV)<br>**in Line 242.**	of musculoskeletal measures with children with CP GMFCS III-V."
**Comment 08**<br>**Line 252-253 & Line 266-267**<br>"(ii) aged between 4.00 to 9.99 years of age at the time of recruitment (<18.00 years of age for Cohort C);"<br>I found this criterion slightly difficult to read. Perhaps it could be rewritten as "(ii) aged from4.00 to 9.99 years (for Cohort A) or from 4.00 to 17.99 years (for Cohort C) at the time of recruitment;" or something similar.<br>Additionally, I am unsure whether the lower age limit for Cohorts B and C should be 4.00 or another value.	The criterion for cohort C is any age less than 18 years, not an age range of 4.00 to 17.99 years. We have amended the sentence for clarity: ii) aged between 4.00 to 9.99 years of age at the time of recruitment (any age less than 18.00 years for Cohort C).
**Comment 09**<br>**Line 273**<br>"... will be retrieved from the NSW/ACT CP Register..."<br><br>The full forms of "NSW" and "ACT" should be provided here but not in **Lines 258–259.**	Thank you, have now amended.
**Comment 10**<br>**Line 278**<br>"... MACS, (CFCS) and..."<br><br>Brackets around CFCS are not necessary here.	Thank you, have now amended.
**Comment 11**<br>**Line 289**<br>"... will involve annual AP pelvis..."<br>The full form of "AP" should be provided here but is not necessary in **Lines 296–297**.	Thank you, have now amended.
**Comment 12**<br>**Line 290-291**<br>"Reimers migration percentage, acetabular index..."<br>Include the abbreviations "MP" and "AI" here, as the abbreviations for other measurements are stated in the same sentence.	Thank you, have now amended.
**Comment 13**<br>**Line 293 & Line 302**<br>"... all AP pelvis X- rays (J Little)."<br>"... for rotational deformity (J Little)."<br><br>I assume "(J Little)" in these two lines refers to one of the co-authors, but what is its purpose?	We agree with the reviewer that it is not necessary and have now removed all mentions of author names throughout main body of manuscript.

**Comment 14**<br>**Line 293-294**<br>"... will include 12 lower limb assessments..."<br><br>Which 12 assessments are the authors referring to?	The ROM is a standardised measure understanding the movement ranges of the hip, knee and ankle. We have added in some more detail: "The physical examination will measure ROM [88] of the hip (flexion, extension, abduction, rotation), knee (extension, flexion) and ankle (dorsiflexion) and this measure has demonstrated good interrater [1, 2], intra-rater[1] and test-rest reliability[3] in children with CP"
**Comment 15**<br>**Line 299**<br>"... X-rays including frontal Cobb angle (Cobb angle),..."<br><br>I believe it is unnecessary to provide "Cobb angle" as the abbreviation for Cobb angle.	Thank you, have now amended.
**Comment 16**<br>**Line 317 & 323**<br>"... will be acquired at T1 and T3, and at T4..."<br>"... at 3 timepoints (T1, T3, T4)..."<br><br>Why is DXA not required at T2?	We thank the reviewer for their comment. Any change in DXA values will be adequately determined be determined by a baseline and 24-month assessment. We do not believe that an addition of a 12-month DXA reading would add significantly to our understanding of the natural history of bone mass accrual in this cohort of children.
**Comment 17**<br>**Line 379**<br>"... by study investigators at CSIRO using..."<br>The full form of CSIRO, "Commonwealth Scientific and Industrial Research Organisation," may need to be provided here.	Thank you, have now amended.

1. McWhirk LB, Glanzman AM. Within-Session Inter-Rater Realiability of Goniometric Measures in Patients with Spastic Cerebral Palsy. Pediatric Physical Therapy. 2006;18(4):262-5 10.1097/01.pep.0000234960.88761.97.
2. Fosang AL, Galea MP, McCoy AT, Reddihough DS, Story I. Measures of muscle and joint performance in the lower limb of children with cerebral palsy. Dev Med Child Neurol. 2003;45(10):664-70 10.1017/s0012162203001245.
3. Klingels K, De Cock P, Molenaers G, Desloovere K, Huenaerts C, Jaspers E, et al. Upper limb motor and sensory impairments in children with hemiplegic cerebral palsy. Can they be measured reliably? Disabil Rehabil. 2010;32(5):409-16 10.3109/09638280903171469.
4. Morris C, Kurinczuk JJ, Fitzpatrick R, Rosenbaum PL. Reliability of the manual ability classification system for children with cerebral palsy. Developmental Medicine & Child Neurology. 2006;48(12):950-3 https://10.1017/s001216220600209x.

VERSION 2 - REVIEW

**Reviewer**	**2**
**Name**	**Chen, Brian Po-Jung**
**Affiliation**	**Chang Gung Memorial Hospital Linkou**
**Date**	**01-Feb-2025**
**COI**	

[1] <u>Response to Comment 01</u>

We thank the reviewer for their comment. The authors acknowledge that it is important to explore the musculoskeletal problems in children with GMFCS level II, however the focus of this study is on the musculoskeletal problems of children with moderate-severe CP, commonly grouped as GMFCS III-V in the literature.

Thanks for your explanation. If so, would the authors consider fine-tuning the title to *"Study Protocol for the Australasian Cerebral Palsy Musculoskeletal Health Network (AusCP MSK): Early Detection of Musculoskeletal Complications in Young Children with Cerebral Palsy at GMFCS Levels III to V"* or something similar? It's absolutely fine to keep the original title. I just personally feel that the terms "moderate" and "severe" do not clearly define the gross motor function of individuals with cerebral palsy.

[2]<u>Response to Comment 03</u>

Musculoskeletal problems often begin in late childhood into early adolescence. The aim of this study is to pick up the earliest biomarkers of musculoskeletal change, hence the decision to recruit children earlier at 4-9 and tracking them over 4 years, when they are 8-13. While we are prospectively assessing musculoskeletal development from age 4, we acknowledge that there might be some important features of musculoskeletal health which contribute to our understanding of early biomarkers before the age of 4 which we can collect retrospectively.

It would be helpful if the authors could integrate this explanation above into the text to give potential readers more insight into how these age ranges were determined.

[3] <u>Response to Comment 16</u>

We thank the reviewer for their comment. Any change in DXA values will be adequately determined be determined by a baseline and 24-month assessment. We do not believe that an addition of a 12-month DXA reading would add significantly to our understanding of the natural history of bone mass accrual in this cohort of children.

Since this is a study protocol manuscript, it would be helpful if the authors could briefly add this explanation to the text to give readers an idea of why T2 (i.e., the 12-month DXA) will be skipped.

===== ===== ===== ===== ===== ===== ===== ===== ===== ===== ===== ===== ===== =====

It is my great pleasure to review this brilliant study protocol. I appreciate the authors' efforts in addressing all queries and revising the manuscript. Aside from the three aforementioned suggestions, I have no further comments. I wish the team success in conducting the *AusCP MSK* study and look forward to reading the study results once they are ready for publication.

VERSION 2 - AUTHOR RESPONSE

Reviewer 2

**Reviewer Comments Round 1**	**Response to Review Round 1**	**Reviewer Comment Round 2**	**Response to Review Round 2**
**Comment 01**<br>**Line 13-18 (ABSTRACT; Page 5)**<br>"... population-based surveillance of children with moderate to severe functional mobility limitations (Gross Motor Function Classification System [GMFCS] levels III-V)..."<br><br>[1] According to a previously published cohort study (n=374) based on the Victorian Cerebral Palsy Register (Soo et al., 2006), the incidence of hip	We thank the reviewer for their comment. The authors acknowledge that it is important to explore the musculoskeletal problems in children with GMFCS level II, however the focus of this study is on the musculoskeletal problems of children with moderate-severe CP, commonly	Thanks for your explanation. If so, would the authors consider fine-tuning the title to "Study Protocol for the Australasian Cerebral Palsy Musculoskeletal Health Network (AusCP MSK): Early Detection of Musculoskeletal Complications in Young Children with Cerebral Palsy	We have now amended the title to reflect the GMFCS levels and also the study design (suggested by the editor). Please see changes in green below:<br><br>Study Protocol for the Australasian Cerebral Palsy Musculoskeletal Health Network (AusCP MSK) Prospective Cohort Study: Early Detection of Musculoskeletal Complications in Young Children with Moderate to Severe Cerebral Palsy (GMFCS III-V).

displacement (i.e., MP > 30%) was 15.1%, 41.3%, 69.2%, and 89.7% for individuals with GMFCS levels II, III, IV, and V, respectively. Although the incidence for individuals with GMFCS level II was low (15.1%), it was not negligible. This proposed study aims to "explore the early biomarkers of, and interactions between, musculoskeletal complications related to CP, including hip displacement..." As the authors stated, "Musculoskeletal complications related to CP for non-ambulant children, including hip displacement, scoliosis and skeletal fragility are often severe and progressive, with a life-long impact." Individuals with GMFCS level II are often overlooked regarding potential hip displacement issues due to their relatively lower risk and higher gross motor function. Were there specific reasons the authors decided to exclude individuals in level II?	grouped as GMFCS III-V in the literature.	at GMFCS Levels III to V" or something similar?<br><br>It's absolutely fine to keep the original title. I just personally feel that the terms "moderate" and "severe" do not clearly define the gross motor function of individuals with cerebral palsy	
**Comment 03**<br>**Line 184-186**<br>" predict the severity and rate of progression of musculoskeletal complications... at 8-13 years"<br>"... on disability outcomes at 8-13 years of age."<br>"... poor quality of life at 8-13 years of age... at 4-9 years of age."	Musculoskeletal problems often begin in late childhood into early adolescence. The aim of this study is to pick up the earliest biomarkers of musculoskeletal change, hence the decision to recruit children earlier at	It would be helpful if the authors could integrate this explanation above into the text to give potential readers more insight into how these age ranges were determined	We thank the reviewer for their comment and have incorporated the following into Line 234:<br><br>Cohort A (n=500) involves the collection of three years retrospective data and subsequent 4-year prospective follow-up (baseline (T1) and 3 annual assessments (T2, T3, T4)) for a range of

, etc.<br><br>**Line 190-195**<br>"... and musculoskeletal development at 1-4 years of age on disability outcomes at 8-13 years of<br>age."<br>"... poor quality of life at 8-13 years of age, related to severity of musculoskeletal complications at 4-9 years of age."<br><br>Could the authors elaborate on how these age ranges (e.g., 1–4 years, 4–9 years, 8–13 years) were determined? Unless I missed some crucial information, it would be helpful if the authors could explain the rationale behind these decisions and their potential relationship with the two time frames the study aims to address.	4-9 and tracking them over 4 years, when they are 8-13. While we are prospectively assessing musculoskeletal development from age 4, we acknowledge that there might be some important features of musculoskeletal health which contribute to our understanding of early biomarkers before the age of 4 which we can collect retrospectively.		musculoskeletal measures with children with CP GMFCS III-V, capturing development from ages 4 to 13 years, providing 7 years of data. This approach enables the identification of early biomarkers of musculoskeletal change, acknowledging that while prospective tracking begins at age 4, important features of musculoskeletal health before this age can be explored through retrospective data collection.
**Comment 16**<br>**Line 317 & 323**<br>"... will be acquired at T1 and T3, and at T4..."<br>"... at 3 timepoints (T1, T3, T4)..."<br><br>Why is DXA not required at T2?	We thank the reviewer for their comment. Any change in DXA values will be adequately determined be determined by a baseline and 24-month assessment. We do not believe that an addition of a 12-month DXA reading would add significantly to our understanding of the natural history of bone mass	Since this is a study protocol manuscript, it would be helpful if the authors could briefly add this explanation to the text to give readers an idea of why T2 (i.e., the 12-month DXA) will be skipped. 	We have added this justification to the end of the DXA section in the measures. Please see addition in green:<br><br>**Dual energy X-ray Absorptiometry (DXA)** scans (GE-Lunar [GE Medical Systems] or Hologic [Hologic Inc, USA]) will be acquired at T1 and T3, and at T4 if a low bone mineral density for age (Z-score <-1.5) at any site is reported at T3. Measures will include age and height matched areal bone mineral density (aBMD; g/cm^2),

	accrual in this cohort of children.		bone mineral content (BMC; grams) at proximal hip, anteroposterior (AP) lumbar spine, total body and lateral distal femur (LDF) [99]. These have been demonstrated to be reproducible in CP samples [100]. Bone mineral apparent density (BMAD, g/cm^3) will be used to estimate volumetric BMD from the AP lumbar spine image. The total radiation exposure to provide all DXA scans at 3 timepoints (T1, T3, T4) would equate to a total radiation exposure of 0.0075 millisieverts (mSv) above normal clinical care. Changes in DXA values will be adequately determined by a baseline (T1), 24-month (T3) and 36 months (T4) assessment. The addition of a 12-month (T2) DXA reading is not expected to significantly enhance the understanding of the natural history of bone mass accrual in this cohort of children.
